# Transcriptome dynamics and allele-specific regulation underlie wheat heterosis at the anthesis and grain-filling stages

**DOI:** 10.1186/s12864-025-11983-2

**Published:** 2025-09-02

**Authors:** Xiaojun Wu, Xiangdong Chen, Ren Wang, Haoquan Wang, Xigui Hu, Yuquan Wang, Gan Li, Na Dong, Tiezhu Hu, Zhengang Ru

**Affiliations:** 1 Wheat Research Center, Henan Institute of Science and Technology, Xinxiang, 453000 China; 2 State Key Laboratory of High-Efficiency Production of Wheat–Maize Double Cropping, Xinxiang, 453000 China; 3 Henan Key Laboratory of Hybrid Wheat, Xinxiang, 453000 China

**Keywords:** *Triticum aestivum* L., Heterosis, Transcriptome, Allele-specific expression, Additive and non-additive expression patterns, *Cis*- and *trans*-regulation, *HSP90.2-B*, Weighted gene co-expression network analysis

## Abstract

**Background:**

As wheat is a globally important staple crop, the molecular regulatory network underlying heterosis in wheat remains incompletely understood. The flag leaf is the primary source of photoassimilates during grain filling and plays a crucial role in yield formation. However, the genetic mechanisms linking flag leaf development to heterosis are still unclear.

**Results:**

Transcriptomic analysis revealed dynamic transcriptional reprogramming during the anthesis to grain-filling transition, with a pronounced expression bias toward superior parental alleles in hybrids. Anthesis-stage non-additive dominance and grain-filling-stage additive enhancement synergistically orchestrated the temporal regulatory shift underlying heterosis. The dominant alleles from the superior parent accounted for more than 60% of the non-additive genes, and the superior parent bias in allele-specific expression ratios progressively increased during development. This highlighted the role of the superior parent as an allelic reservoir. *Cis*-regulatory variations primarily contributed to additive effects, whereas *cis*×*trans* interactions were the primary regulatory driver of positive overdominance. Notably, weighted co-expression network analysis identified *HSP90.2-B* as a putative heterosis-related gene, whose coordinated overexpression with AP2/ERF transcription factors provides valuable insights for elucidating the molecular basis of yield heterosis.

**Conclusions:**

This study establishes two complementary models to decode the molecular regulation of heterosis in wheat. The “dual-engine” model demonstrates stage-specific gene expression patterns: non-additive effects predominantly drive early growth vigor during the anthesis stage, whereas additive expression patterns stabilize grain development and yield-related traits at the grain-filling stage. The “two-phase regulatory shift” model captures the dynamic temporal progression of heterotic regulation, evolving from *trans*-regulation-driven plastic responses at the anthesis stage to *cis*-regulation-mediated homeostatic control at the grain-filling stage. Importantly, the preferential coupling between *cis*-regulation/additive and *trans*-regulation/non-additive expression provides molecular evidence supporting the complementary nature of the models. We further identified developmentally specific modules (the anthesis-stage Red module and grain-filling-stage Brown module) with their core regulatory networks through weighted gene co-expression network analysis. These findings preliminarily characterize the multi-layered cooperative networks regulating heterosis development, potentially offering valuable theoretical clues for deciphering the molecular mechanisms underlying wheat heterosis.

**Supplementary Information:**

The online version contains supplementary material available at 10.1186/s12864-025-11983-2.

## Introduction

Compared with genetically distinct parental lines, first-generation hybrids (F_1_) present superior performance in various traits, with performance traits including growth vigor, viability, fertility, adaptability, and yield [1, 2]. Heterosis is fundamental in crop genetic breeding and has become an essential driver of agricultural development. For example, the successful large-scale cultivation of hybrid maize and rice has been vital for ensuring global food security [3, 4]. Wheat (*Triticum aestivum* L.) is one of the most commonly consumed staple crops. It provides approximately 20% of a wheat consumer’s total caloric intake and serves as the primary food source for 30% of the global population [5]. Research has demonstrated that hybrid wheat results in substantial yield improvements over parental lines, with productivity increasing 10–54.3% [6, 7]. However, hybrid wheat currently accounts for less than 1% of the global area dedicated to wheat cultivation [4]. Therefore, an in-depth examination and commercial application of wheat heterosis are important for ensuring global food security and preserving ecological balance.

Genetic models, such as dominance, overdominance, and epistasis, have been tested to explain heterosis [1, 8, 9], but a single model currently does not fully explain this complex phenomenon. Owing to advances in omics technologies, notably transcriptomics, heterosis can now be investigated at the molecular level. RNA sequencing (RNA-seq) reveals differences in gene expression between hybrids and parental lines [10, 11], whereas allele-specific expression (ASE) analysis dissects parental allele imbalances in hybrids [12, 13]. ASE has been observed in numerous species, including *Arabidopsis* [14], maize [15–18], rice [13, 19, 20], and wheat [21], highlighting the evolutionarily conserved nature of this trait.

ASE is a complex process regulated by epigenetics and genetic variations in response to developmental cues and environmental conditions [22]. A genome-wide analysis of ASE genes in apple revealed that insertions of transposable elements in the upstream regulatory regions of genes play a significant role in ASE regulation [23]. In rice, loss-of-function nonsense single-nucleotide polymorphism (SNP) and insertion/deletion (indel) mutations within the coding sequences of allele-specific expressed genes (ASEGs) lead to premature protein truncation, functional loss, or nonsense-mediated mRNA decay [13]. Notably, most ASEGs in rice hybrids are associated with DNA methylation patterns, and the ASEGs are negatively correlated with allele-specific CHG methylation [24]. Similarly, ASEGs in potato are likely to be negatively regulated by allele-specific CHH methylation, which is consistent with the repressive role of methylation in gene expression [25]. These studies demonstrate that ASE occurs in a multilayered fashion, which includes transcriptional control, posttranscriptional processing, and translational regulation. ASEGs displaying consistent expression bias toward one of the parental alleles in all tissues or under all conditions may confer partial-to-complete dominance effects on phenotypic traits, whereas ASEGs with directional shifts in different tissues or under different conditions could drive overdominance [13].

Despite the potential for heterosis in wheat and rice, its application in wheat remains limited for two main reasons. First, heterotic groups in wheat have not yet been sufficiently investigated. Second, the added complexity of working with polyploid genomes, the inefficiency of hybridization techniques, and the lagging development of molecular breeding tools make identifying superior wheat hybrids challenging [26]. Little is known about the molecular mechanisms of wheat heterosis. Preliminary studies include those of Liu et al. [21], who used transcriptomics, gene expression patterns, and ASE analysis in hybrid seedlings and spike tissues to elucidate the molecular regulatory mechanisms underlying heterosis in different tissues. The results revealed that the expression of genes related to photosynthesis and carbon fixation was upregulated in seedling tissues, whereas transcription factor activities were significantly enriched in spike tissues, highlighting the specific contribution of metabolic pathways and transcriptional regulation to hybrid dominance in different tissues. Systematic investigations integrating transcriptomics and ASE technologies are currently lacking, which limits our ability to fully understand the regulatory networks involved at the molecular level. This underscores the urgent need for integrated studies encompassing various developmental stages and tissue types to explain the genetic and regulatory mechanisms underlying heterosis in wheat.

The period from anthesis to grain maturation determines the quality of wheat grain development and yield [27, 28]. The flag leaf is the primary source of photoassimilates for grains and contributes over 50% of the carbon photoassimilates to the overall yield [29, 30]. The central role of flag leaves in determining yield suggests that they influence heterotic phenotypes via regulatory pathways associated with hybrid vigor. Comparative transcriptomics analyses revealed that the flag leaves of hybrid plants exhibit significantly different gene expression patterns than those of the parental lines do, and these differences have an impact on heterotic yield-related traits [31, 32]. Furthermore, wheat transcriptionally reprograms itself in a stage-specific manner between the anthesis and grain-filling stages, with flag leaves undergoing particularly dynamic transcriptional shifts [33]. When plants transition from anthesis to grain filling, the physiological function of flag leaves shifts from photosynthetic dominance to nutrient remobilization. The expression profiles during this phase are highly dynamic and reflect the metabolic reorganization associated with heterosis and spatiotemporal regulation via allele-specific complementation. The strong correlation between stage-specific transcriptional dynamics and phenotypic traits highlights the importance of this period when examining flag leaf-mediated heterosis mechanisms.

This study investigated the molecular regulatory network underlying heterosis in wheat via genome-wide ASE analysis of the superior hybrid BC98 and its parental lines Bainong 4199 and CL0438 via flag leaf transcriptome data from the anthesis and grain-filling stages. By systematically investigating the expression dynamics of heterosis-related genes at different developmental stages and the characteristics of ASE and *cis-* and *trans-*regulatory mechanisms, we constructed a multidimensional regulatory gene expression model to understand heterosis development in wheat flag leaves. Weighted gene co-expression network analysis (WGCNA) was used to identify important co-expression modules that revealed coordinated regulatory patterns of heterosis-associated genes and their involvement in various biological pathways. These findings allow us to understand the regulatory mechanisms underlying yield-related heterosis traits in wheat.

## Materials and methods

### Plant materials

This study used the wheat hybrid BC98, the maternal cultivar Bainong 4199 (BN4199, a commonly cultivated cultivar in Henan Province, China), and the paternal line CL0438 for the experiments. On the basis of our team’s previous research on heterotic wheat group classification [34], BN4199 belongs to the Huang-Huai group and presents superior agronomic traits. The Chilean-sourced introgression line CL0438, when crossed with Huang-Huai group lines, demonstrated significant heterosis. The plants were cultivated in experimental fields located in Xinxiang city, Henan Province (113°55′E, 35°18′N) during autumn 2023 with regular water and fertilizer management. Hybrid seeds of BC98 were generated through strictly controlled manual cross-pollination. The maternal parent BN4199 was emasculated manually prior to anther dehiscence: (1) Spikelets were opened using fine forceps; (2) Anthers were carefully removed without damaging pistils; (3) Emasculated spikes were immediately bagged with parchment paper to prevent accidental pollination. Pollen from the paternal line CL0438 was collected at anthesis and applied to BN4199 stigmas 48 h after emasculation. Cross-pollinated spikes were rebagged until seed maturation. Hybrid purity was confirmed through a dual-validation system: (1) Field-based phenotypic screening: Non-hybrid plants were excluded based on distinct differentiation in vegetative growth traits (plant height, tillering pattern, etc.) during early developmental stages; (2) Post-harvest kernel phenotyping: Secondary verification was performed using mature kernels harvested from the same individual plants previously subjected to flag leaf sampling, with kernel color and morphology evaluated for significant divergence relative to parental lines.

### Sample preparation, transcriptome sequencing, and data analysis

A total of 18 samples were analyzed, comprising three genotypes (the parental lines BN4199 and CL0438, and their hybrid BC98) at two developmental stages (anthesis (AN) and grain-filling (GF)), with three independent biological replicates per genotype per stage. Each biological replicate consisted of a pool of flag leaves from 10 individual plants grown under uniform field conditions. Developmental stages were meticulously synchronized, with AN defined as 50% main spike flowering and GF as 14 days post-flowering, while sampling windows were controlled within < 3 days across genotypes.

Total RNA was extracted via TRIzol^®^ reagent (Invitrogen, Carlsbad, CA, USA) and assessed for quality via a NanoPhotometer^®^ spectrophotometer (IMPLEN, Westlake Village, CA, USA), Agilent 2100 Bioanalyzer (Agilent Technologies, Santa Clara, CA, USA), and agarose gel electrophoresis. UMI-labeled paired-end libraries were constructed via the NEBNext Ultra RNA Library Prep Kit (Ipswich, MA, USA) and quantified with a Qubit^®^ 2.0 fluorometer (Invitrogen). The fragment size of the libraries was validated via an Agilent 2100 Bioanalyzer. Finally, all the libraries were paired-end sequenced (2 × 150 bp) on the Illumina NovaSeq™ 6000 platform (Illumina, San Diego, CA, USA) at Novogene Co., Ltd. (Beijing, China).

We used Novogene’s proprietary pipeline to preprocess the raw sequencing reads to remove adapters, low-quality reads (defined as reads containing > 50% bases with Phred ≤ 20), and reads with ambiguous bases (N). Clean reads were aligned to the Chinese Spring reference genome (IWGSC_RefSeq v2.1) via HISAT2 (v2.2.1) [35]. Transcript assembly was performed using StringTie v2.2.1 with default parameters to reconstruct transcripts from clean reads. To accurately quantify the expression level of individual homoeologues (A-, B-, and D-genome specific copies), only reads that were uniquely mapped to the reference genome were retained for gene expression quantification and subsequent differential expression analysis. Reads aligning equally well to multiple locations were excluded from gene-level counts to avoid cross-mapping artifacts and ensure locus-specific expression measurement. This filtering resulted in 86.4% of the clean reads being uniquely mapped and utilized for expression quantification.

Differential expression analysis was performed on the NovoMagic cloud platform (Novogene, Beijing, China). Unnormalized read counts were used as input for DESeq2 [36] to identify differentially expressed genes (DEGs) with thresholds of |log^2^(fold change)| ≥ 1 and adjusted *p* value < 0.05. For visualization purposes, gene expression levels were normalized as fragments per kilobase of transcript per million mapped reads (FPKM).

### Classification of differential gene expression patterns

Differential gene expression analysis was conducted via the DESeq2 package (v1.40.2) within the R statistical environment (v4.3.1), and the ​​Benjamini‒Hochberg method (also known as the false discovery rate (FDR))​​ was used to control for Type I errors. Genes with an ​​adjusted *p* value (*q* value) < 0.05​​​ were classified as significantly differentially expressed. To identify regulatory patterns in the ​​F_1_ hybrids​​, their expression levels were compared with the ​​midparent value (MPV)​​. The genetic regulatory categories were defined as follows: (1) additive expression: genes showing no significant deviation between F_1_ and MPV (*q* > 0.05); and (2) non-additive expression: genes exhibiting significant differences between F_1_ and MPV (*q* < 0.05). The genes in category (2) were further classified into the following categories: dominance (high-parent dominance): F_1_ expression showing no significant difference from the high-expressing parent (*q* > 0.05) but was significantly elevated compared with the low-expressing parent (*q* < 0.05); dominance (low-parent dominance): F_1_ expression aligned with the low-expressing parent (*q* > 0.05) but was significantly reduced relative to the high-expressing parent (*q* < 0.05); overdominance/underdominance: genes where F_1_ expression was significantly higher (overdominance) or lower (underdominance) than that of the parental lines.

### SNP identification and allele-specific expression analysis

SNP detection and allele-specific expression (ASE) analysis were performed exclusively using reads uniquely mapped to the reference genome, ensuring that identified SNPs and allelic biases could be assigned to specific homoeologous loci. The transcriptome data were analyzed to identify SNPs between the parental lines BN4199 and CL0438 with rigorous quality control measures. Both parental reads were independently aligned to the reference genome using identical HISAT2 parameters as those applied to hybrid samples, ensuring mapping consistency. RNA-seq replicates were processed separately to maintain biological independence, with allele counts aggregated only after binomial testing. The resulting parental BAM alignment files (generated by HISAT2) were processed using Picard tools (v1.96) and SAMtools (v0.1.18) [37] for duplicate marking and BAM file sorting. Potential reference mapping bias was controlled by excluding sites exhibiting > 2-fold reference allele skew in hybrids, following the approach of Castel et al. (2015) [38]. SNP detection was performed via GATK4 [39] with stringent filtering criteria: (1) unique genome alignment of parental reads (MAPQ ≥ 20 and NH: i:1 flag), (2) consensus base differences between parents at SNP loci, (3) minimum read depth of 10 at SNP sites, and (4) exclusion of indels and SNPs in extreme depth regions (top/bottom 5% of depth distribution). Genes harboring at least one SNP site that passed all the above quality filters (in either parent) and achieved sufficient read depth (≥ 10) in the hybrid samples were defined as “SNP-qualified genes” and subjected to ASE analysis. For ASE analysis, allele-specific read counts at SNP loci in hybrids were obtained using GATK4’s ASEReadCounter, followed by binomial testing (α = 0.05, null hypothesis allele ratio = 0.5). Sites showing significant deviations (FDR-adjusted *p* < 0.05) were classified as ASE events.

Genes were classified into three allelic expression patterns based on hybrid SNP read ratios. Monoallelic expression required both (1) significant deviation from the expected 0.5 ratio (binomial test, FDR-adjusted *p* < 0.05) and (2) an allelic ratio ≥ 0.95. Preferential expression required significant deviation (FDR-adjusted *p* < 0.05) coupled with either ≥ 0.67 or ≤ 0.33 allelic ratio (representing ≥ 2:1 transcript bias toward either parental allele). Biallelic expression was defined by non-significant deviation (FDR-adjusted *p* ≥ 0.05) combined with an allelic ratio strictly between 0.33 and 0.67 (indicating < 2:1 bias).

### Dissecting *cis*- and *trans*-regulatory divergence

The contributions of *cis*- and *trans*-regulation were analyzed by comparing the ASE ratios in the hybrids with the expression divergence of the parental lines, following established methods [16, 40]. The key steps included the following:

1. Global expression divergence (P): parental differences (log_2_(CL0438/BN4199)) were quantified via binomial exact tests (FDR-adjusted *q* < 0.05).
2. *Cis*-regulatory effects (H): the ASE imbalance in the hybrids (log_2_(CL0438_BC98_/BN4199_BC98_)) was assessed via binomial tests (FDR-adjusted *q* < 0.05).
3. Transregulatory effects (T): These effects were derived as T = P – H and tested via Fisher’s exact tests (FDR-adjusted *q* < 0.05).

The genes were categorized into five regulatory classes: (1) *cis*-only: significant P and H (*q* < 0.05) and nonsignificant T; (2) *trans*-only: significant *P* and T (*q* < 0.05) and nonsignificant H; (3) *cis*-*trans* interaction: significant P, H, and T (*q* < 0.05); (4) conserved: nonsignificant P, H, and T; and (5) ambiguous: unclassifiable patterns lacking clear biological interpretation.

The *​cis*-*trans* interaction category was further divided into three subtypes: (1) synergistic (*cis* + *trans*): significant *​P*, ​H, and ​T, and *cis*- and *trans*-regulation jointly enhance the expression of the same allele; (2) antagonistic (*cis* × *trans*): significant ​P, ​H, and ​T, and regulatory forces drive opposing alleles; and (3) compensatory: significant ​H and ​T with nonsignificant ​P, and regulatory divergence counterbalances species-level expression differences.

### Identification of gene co-expression modules

Co-expression networks were constructed using the WGCNA package (v1.72) in R (v4.3.1) based on log_2_-transformed FPKM values [41]. To ensure robust module identification given the limited sample size (*n* = 18), the following reliability-enhancing strategies were implemented: (1) A soft-thresholding power (β = 11) was selected as the minimal value achieving scale-free fit R² >0.85. (2) Conservative module detection and merging parameters were applied (minModuleSize = 30, mergeCutHeight = 0.25) to minimize spurious clustering. (3) Eigengene correlations with sample groups were quantified and visualized in heatmaps. Gene module assignment used dynamic tree-cutting of hierarchical clustering dendrograms. Intra-modular connectivity (kWithin) was calculated to identify high-connectivity hub genes for downstream analyses. To rigorously assess the association of co-expression modules with heterosis dynamics, we performed a two-way ANOVA on module eigengene (ME) values. The model included Genotype (BN4199, CL0438, BC98) and Developmental Stage (AN, GF) as fixed factors, and their interaction term (Genotype × Stage). Analyses were conducted in R v4.3.1 using the aov() function. Tukey’s Honestly Significant Difference (HSD) post hoc tests were applied only if a significant main effect (*p* < 0.05) was observed without a significant interaction (*p* > 0.10). Modules exhibiting significant interaction effects (*p* < 0.01) were selected for downstream functional analyses.

Genes exhibiting ≥ 2-fold expression changes in the target modules were functionally annotated on the basis of the UniProt database to define their molecular roles. High-confidence protein‒protein interaction (PPI) networks were constructed via STRING (https://string-db.org/; edge weight ≥ 0.6). The hub genes (top 10% connectivity nodes) were identified via Cytoscape (v3.7.2) and considered to perform critical regulatory functions. Gene Ontology (GO) and Kyoto Encyclopedia of Genes and Genomes (KEGG) pathway enrichment analyses (FDR < 0.05) were performed on the module genes via NovoMagic (https://magic.novogene.com/) to reveal the biological processes and metabolic pathways involved in stage-specific network dynamics.

## Results

### Phenotypic evaluation of hybrid and parental lines

**Fig. 1:**
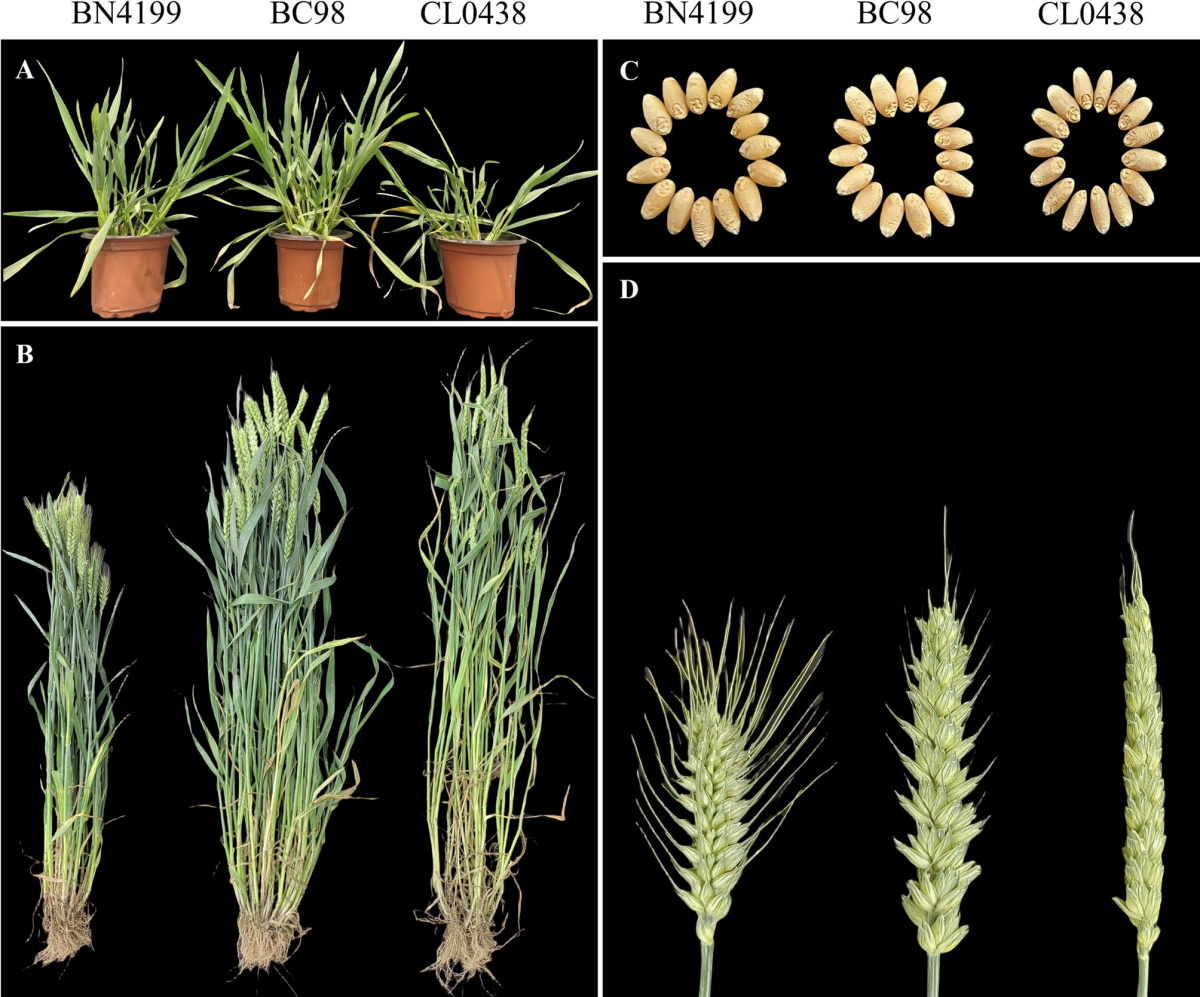
Phenotypic evaluation of the wheat hybrid BC98 and its parental lines. The plant lines in each figure are displayed in the following order: BN4199, BC98, and CL0438. (**A**) Heterotic phenotypes at the seedling stage. (**B**) The heterotic phenotypes at the adult plant stage. (**C**) Heterotic phenotypes of the grains. (**D**) The heterotic phenotypes of the spikes

Phenotypic assessments revealed enhanced hybrid vigor in hybrid BC98, characterized by increased seedling vigor and biomass accumulation at the seedling and adult stages (Fig. 1A, B). Adult BC98 exhibited superior agronomic traits, which included higher spike numbers and grain yields per plant (Table 1). Notably, compared with both parental lines, BC98 presented significantly more spikelets per spike, demonstrating substantial mid-parent heterosis (MPH) and better-parent heterosis (BPH; *p* < 0.01) (Table 1; Fig. 1C, D). Additional heterotic traits included plant height, flag leaf area, spike length, and weight per 1000 grains (Table 1). These results confirmed the robust heterosis of BC98, particularly with respect to yield-related traits. Additionally, for most yield-related heterosis traits, BC98 surpassed the parental line BN4199 (a widely cultivated commercial variety), highlighting the potential of BC98 in agricultural applications.

**Table 1:** Evaluation of heterosis for yield-related agronomic traits in the hybrid BC98

Trait	BN4199 ^m^	CL0438 ^m^	BC98 ^m^	MPH (100%)	BPH (100%)
Plant height (cm)	78.3 ± 2.81 ^c^	112.0 ± 3.85 ^a^	100.6 ± 2.61 ^b^	5.75 **	−10.18 **
Flag leaf area (cm^2^)	31.6 ± 2.28 ^c^	49.2 ± 4.76 ^a^	45.9 ± 4.20 ^b^	13.16 *	−6.72
Spike number	10.8 ± 1.78 ^b^	9.2 ± 1.97 ^b^	15.2 ± 2.82 ^a^	52.00 **	40.74 **
Spike length (cm)	9.3 ± 0.34 ^c^	13.2 ± 0.75 ^a^	12.6 ± 0.54 ^b^	12.03*	−4.49
Spikelet number	21.1 ± 1.44 ^c^	22.5 ± 1.36 ^b^	24.0 ± 1.31^a^	10.09 **	6.51 **
Grain yield per plant (g)	23.0 ± 2.47 ^b^	15.3 ± 3.22 ^c^	34.1 ± 3.93 ^a^	78.38 **	48.40 **
Thousand-grain weight (g)	44.1 ± 1.84 ^a^	30.7 ± 1.41 ^b^	40.4 ± 0.69 ^a^	8.06 *	−8.28

^**^Indicates significant differences at *p* < 0.01; * indicates significant differences at *p* < 0.05

^m^Values are means ± standard deviations. MPH, mid-parent heterosis; BPH, better-parent heterosis

^a-c^Different letters within a column indicate significant differences (*p* < 0.05, Duncan's test)

### Transcriptome sequencing of hybrid wheat and parental lines

This study performed transcriptome sequencing of flag leaves from hybrid BC98 and the parental lines BN4199 and CL0438 during the AN and GF stages via the Illumina NovaSeq 6000 platform. A sequencing depth of more than 10 Gb per sample was obtained (total of 18 samples, including biological triplicates), and 66.4–104.3 million high-quality reads per sample were aligned to the Chinese Spring wheat reference genome (IWGSC RefSeq v2.1), resulting in an overall mapping rate of 86.8%, with 86.4% of clean reads being uniquely mapped (Table S1). The biological replicates were highly consistent (average Pearson *r* = 0.95) (Figure S1). After filtering, 92,845 high-confidence protein-coding genes were identified (Table S2). Among the 47,838 expressed genes (FPKM ≥ 1), the expression levels were categorized as high (FPKM ≥ 50, 4.3% or 2,068 genes), moderate (20 ≤ FPKM < 50, 7.5% or 3,604 genes), and low (FPKM < 20, 88.2% or 42,568 genes). At the AN stage, the paternal line CL0438 exhibited the broadest expression profile (48,835 genes with FPKM ≥ 1), while during the GF stage, the hybrid BC98 showed the most extensive expression spectrum (48,805 genes with FPKM ≥ 1).

### Differences in expression between hybrid and inbred parents

**Fig. 2:**
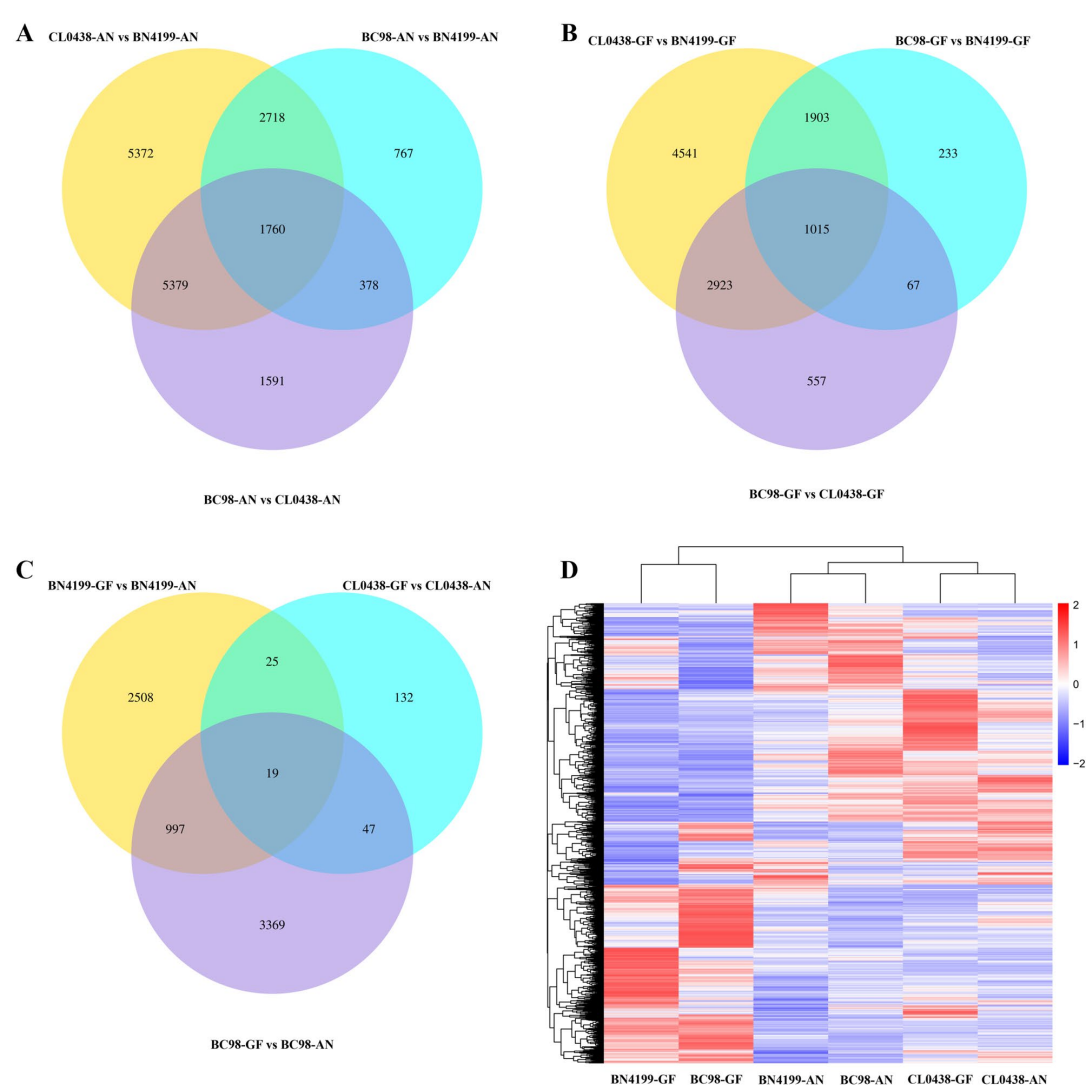
Venn diagram comparisons and hierarchical clustering analysis of differentially expressed genes (DEGs) among the plant lines. (**A**) Venn diagram comparison of the DEGs at the anthesis (AN) stage. (**B**) Venn diagram comparison of the DEGs at the grain-filling (GF) stage. (**C**) Venn diagram comparison of the DEGs between the AN and GF stages. (**D**) Hierarchical clustering analysis of the DEGs among the plant lines. The color scale in (**D**) represents the log_10_(FPKM + 1) values, with red indicating relatively high expression and blue indicating relatively low expression

Comparative transcriptomic analysis revealed dynamic expression divergence between the hybrid BC98 and its parental lines at the AN and GF stages. At the AN stage, 15,229 DEGs (*|*log_2_FC*|* ≥ 1, *p* < 0.05) were identified between the parents (68.6% of the total number of genes; 27.3% upregulated, 41.3% downregulated) (Fig. 2A, Table S3). The hybrid BC98 exhibited a BN4199-biased expression profile, with fewer upregulated DEGs relative to the parental line BN4199 (5,623 DEGs; 17.3% upregulated) than the parental CL0438 (9,108 DEGs; 20.2% upregulated). At the GF stage, the number of DEGs identified between the parental lines decreased (10,328 parental DEGs; 46.7% of total genes), but the upregulated/downregulated gene ratios increased (Fig. 2B, Table S3). Notably, 46.0% of the DEGs identified between the parental lines at the AN stage and 37.9% at the GF stage overlapped with those identified between BC98 and CL0438, whereas only 29.4% at the AN stage and 28.1% at the GF stage overlapped between BC98 and BN4199 (Fig. 2C, Table S3). Hierarchical clustering analysis confirmed the hybrid BC98’s expression bias toward the parental BN4199 (Fig. 2D).

A comparative analysis across the developmental stages revealed distinct expression dynamics in the hybrid BC98 and its parental lines. Stage-specific DEGs were identified in BC98 (4,432 DEGs), BN4199 (3,549 DEGs), and CL0438 (223 DEGs) when the AN and GF stages were compared (Fig. 2C, Table S3). The hybrid BC98 shared 1,016 DEGs with the parental line BN4199 but only 66 with the parental CL0438. Notably, TraesCS4B02G178500 (a VQ domain-containing protein), which was upregulated in BC98 at the GF stage (Fig. 2C), delays leaf senescence by suppressing WRKY75 [42]. Further comparison of the two development stages revealed 3,549, 223, and 4,432 DEGs in BN4199-GF vs. BN4199-AN, CL0438-GF vs. CL0438-AN, and BC98-GF vs. BC98-AN, respectively (Table S3).

A Venn diagram revealed that 1,088 genes were differentially expressed between the two development stages in at least two genotypes (Fig. 2C). GO enrichment analysis revealed that these DEGs were significantly enriched in multicellular organismal processes (GO:0032501) and nutrient reservoir activity (GO:0045735) (Table S4). Multiple organismal processes (GO:0032501) involve primarily serine/threonine protein kinases, which use phosphorylation to regulate signal transduction and tissue differentiation [43]. Moreover, nutrient reservoir activity (GO:0045735) was enriched with germin-like protein genes involved in cell wall remodeling, starch‒sugar metabolic flux regulation, and responses to biotic/abiotic stresses [44]. Notably, the GO analysis revealed that the hybrid BC98 was more similar to the parental line BN4199 in terms of developmental signaling regulation and metabolic characteristics. This pattern was further confirmed through hierarchical clustering of the 1,088 DEGs (Figure S2).

Collectively, these gene expression results closely aligned with the phenotypic observations. Specifically, the parental lines BN4199 and CL0438 presented strong complementary traits, with BN4199 serving as the high-value parent for critical yield-related characteristics, such as the weight per thousand grains and single-plant yield. These results highlight the central role of BN4199 in increasing the yield of hybrid plants.

### Dynamic changes in differential gene expression patterns affect heterosis

**Fig. 3:**
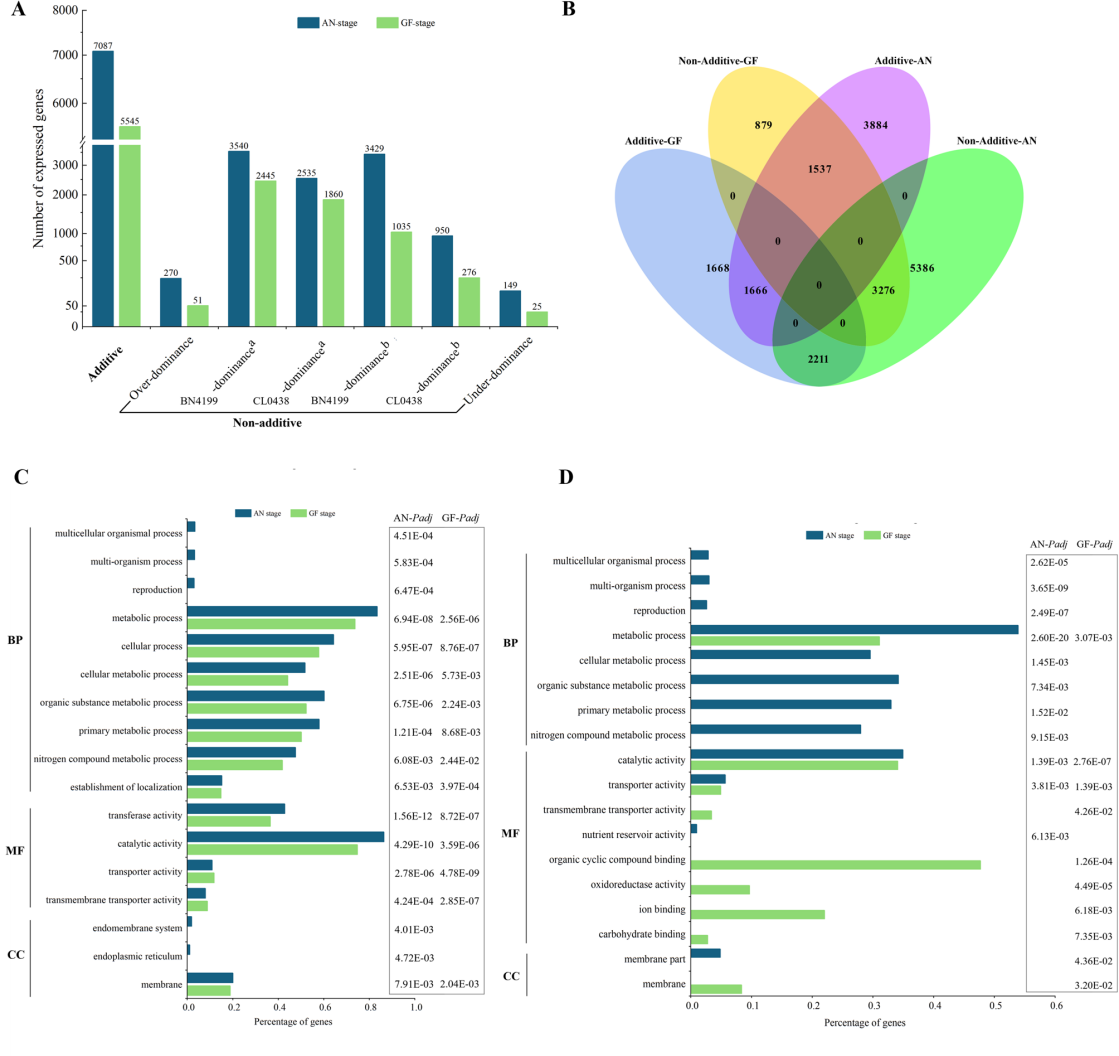
Comparative analysis and functional enrichment analysis of additive and non-additive genes in hybrid plants across developmental stages. (**A**) Distribution of additive and non-additive genes at the anthesis (AN) and grain-filling (GF) stages. ^a^ denotes high-parent dominance, and ^b^ indicates low-parent dominance. (**B**) Venn diagram depicting inheritance pattern transitions between the AN and GF stages. (**C**) Gene Ontology enrichment of the additive genes. (**D**) Stage-specific Gene Ontology enrichment patterns of BN4199-dominant genes in the hybrids at the AN and GF stages

To elucidate the transcriptomic divergence between the F_1_ hybrids and their parental lines, we classified DEGs from two critical developmental stages into additive and non-additive gene expression patterns using the MPV as the reference baseline. At the AN stage, 7,087 genes (39.5%) presented additive expression, which declined numerically but increased proportionally to 5,545 genes (49.3%) at the GF stage. In contrast, the non-additive genes dominated at the AN stage (10,873 genes; 60.5%) but decreased numerically and proportionally at the GF stage (5,692 genes; 50.7%) (Fig. 3A and Tables S5, S6). The non-additive genes were further categorized into parental dominant (high/low-parent dominance) and over/underdominant types. At the AN stage, parental dominance accounted for 96.1% of the non-additive genes (6,969 BN4199-dominant genes vs. 3,485 CL0438-dominant genes), whereas the over- and underdominant types were rare (2.5% and 1.4%, respectively). At the GF stage, parental dominance remained predominant (98.7%), with the BN4199-dominant genes consistently outnumbered the CL0438-dominant genes (61.1% vs. 37.5%). The number of overdominant and underdominant genes further decreased to 51 (0.9%) and 25 (0.4%), respectively. Notably, the BN4199-dominant genes accounted for more than 60% of the non-additive genes across both developmental stages, indicating that their alleles likely serve as key drivers of heterosis by regulating critical pathways.

The non-additive genes presented greater developmental stage-dependent expression changes, declining by 47.9% (5,181 genes) from the AN stage to the GF stage, compared with only 21.8% for the additive genes (Fig. 3A and Tables S5, S6). Consequently, the proportion of additive genes increased from 39.5% at the AN stage to 49.3% at the GF stage, indicating a shift from non-additive interactions governing early growth to additive genetic complementation driving grain filling. Examining the degree of conservation in the expression patterns between the developmental stages revealed distinct dynamics between the non-additive and additive gene expression patterns. At the GF stage, 30.1% of the non-additive genes (3,276 genes) maintained the same non-additive expression patterns, whereas 20.3% (2,211 genes) shifted to additive patterns (Fig. 3B). In contrast, the additive genes presented lower stability, with only 23.5% (1,666 genes) retaining their original expression patterns and 21.7% (1,537 genes) transitioning to non-additive expression patterns. These findings demonstrated that gene expression patterns display distinct growth stage-specific adaptability in response to developmental and environmental cues. These observations were also consistent with prior studies of transcriptional plasticity in rice hybrids [45].

### Functional enrichment analysis of genes whose expression patterns are linked to heterosis

We performed comprehensive GO enrichment analyses to investigate the molecular functions and biological contributions of additive and non-additive genes to heterosis. Among the additive genes, 1,394 genes (19.7% of the 7,087 additive genes) at the AN stage and 1,013 genes (18.3% of the 5,545 additive genes) at the GF stage were significantly enriched (adjusted *p* < 0.05) in biological processes (BP), molecular functions (MF), and cellular components (CC) (Fig. 3C, Table S7). Among the genes involved in BP, metabolic processes (GO:0008152; 83.5% at the AN stage and 73.7% at the GF stage) and cellular processes (GO:0009987; 64.3% and 57.8%) were enriched at both developmental stages, whereas reproductive processes (GO:0022414; 3.1%) were specifically enriched at the AN stage. Among these processes, the additive genes involved in core metabolic and cellular pathways were consistently enriched at both stages, including primary metabolic processes (GO:0044238; 58.0% vs. 50.1%), cellular metabolism (GO:0044237; 51.8% vs. 44.2%), organic substance metabolism (GO:0071704; 60.2% vs. 52.3%), protein metabolism (GO:0019538; 38.6% vs. 29.3%), establishment of localization (GO:0051234; 15.2% vs. 14.9%), catalytic activity (GO:0003824; 86.4% vs. 74.8%), transporter activity (GO:0005215; 11.0% vs. 11.8%), and membrane-associated components (GO:0016020; 20.1% vs. 19.0%). These results indicate that additive genes primarily sustain basal cellular metabolism and macromolecule biosynthesis, which aligns with previous heterosis studies [16]. Stage-specific enrichment patterns reflect the dynamic nature of these functional adaptations, and the high proportion of genes involved in catalytic activity suggests that they play essential regulatory roles. KEGG enrichment revealed stage-coordinated metabolic network remodeling (Table S8). At the AN stage, characteristic pathways including starch and sucrose metabolism (ko00500), plant-pathogen interaction (ko04626), diterpenoid biosynthesis (ko00904), and alpha-linolenic acid metabolism (ko00592) collectively supported reproductive organ development and environmental adaptation. Transitioning to the GF stage, the pathway network markedly shifted toward reproductive optimization, with flavonoid biosynthesis (ko00941), plant hormone signal transduction (ko04075), ABC transporters (ko02010), and circadian rhythm (ko04712) dominating resource allocation efficiency. This metabolic remodeling from “defense priming” at the AN stage to “reproduction priority” at the GF stage was sustained by conserved pathways, such as phenylpropanoid biosynthesis (ko00940), homologous recombination (ko03440), and starch and sucrose metabolism (ko00500), maintaining basal stress resilience and genomic stability, while stage-enhanced transport and signaling pathways enabled precise developmental transitions. These KEGG patterns aligned closely with GO enrichment of additive genes, corroborating the centrality of core metabolism. Notably, the significantly enriched amino acid biosynthesis pathway (GO:0019538) at the AN stage and the enhanced nitrogen metabolism (Ko00910) at the GF stage worked synergistically to maintain protein metabolic continuity. Furthermore, the enrichment of ABC transporters (Ko02010) at the GF stage corresponded functionally to the elevated activity of “establishment of localization” (GO:0051234) identified in GO analysis. This coordinated regulation demonstrates that additive genes maintain heterosis through a highly active metabolic-catalytic homeostasis system that forms a fundamental support network, while developmental stage-dependent pathway reorganization facilitates the dynamic transition of heterosis from reproductive development at anthesis to assimilate partitioning during grain filling, thereby providing the molecular basis for achieving both high and stable yields.

Among the non-additive genes, GO analysis revealed 2,175 (20.0% of the 10,873 genes) and 930 (16.3% of the 5,692 genes) significantly enriched genes at the AN and GF stages, respectively (Table S7). Among the BN4199-dominant genes, we observed unique patterns in those belonging to BP: metabolic processes (GO:0008152; 53.9% at the AN stage vs. 31.1% at the GF stage) and reproductive processes (GO:0022414; 2.6% exclusively at the AN stage) (Fig. 3D). MF analysis revealed that catalytic activity (GO:0003824; 34.9% vs. 34.1%) and transporter activity (GO:0005215; 5.7% vs. 4.9%) were enriched. At the AN stage, the stage-specific MF terms included nutrient reservoir activity (GO:0045735; 1.0%), and at the GF stage, they included organic cyclic compound binding (GO:0097159; 47.7%), oxidoreductase activity (GO:0016491; 9.7%), ion binding (GO:0043167; 22.0%), and carbohydrate binding (GO:0030246; 2.8%). In contrast, the CL0438-dominant genes were not enriched and included primarily catalytic activity (GO:0003824; 39.5%) at the GF stage (Table S7). These findings demonstrate that BN4199-dominant alleles contribute disproportionately to functional GO terms. This observation also suggests that these BN4199-dominant alleles play stage-specific regulatory roles in heterosis.

The KEGG pathway analysis of the GF-stage BN4199-dominant genes revealed several key pathways contributing to parental dominance (Table S9), which included metabolic pathways (ko01100), endoplasmic reticulum protein processing (ko04141), carbon metabolism (ko01200), plant‒pathogen interaction (ko04626), glutathione metabolism (ko00480), phenylpropanoid biosynthesis (ko00940), and circadian rhythm (ko04712). Notably, we identified many functionally characterized genes associated with yield and stress resilience, likely contributing to BC98 heterosis. These genes included *RBCS* (Rubisco small subunit), which is involved in photosynthetic carbon assimilation [46]; *SPS* (sucrose-phosphate synthase), which regulates sucrose biosynthesis [47]; *IPT* (isopentenyltransferase), which is a cytokinin biosynthesis rate-limiting enzyme that enhances stress tolerance and yield [48]; *CYP90B1*, which encodes a brassinosteroid biosynthesis enzyme critical for development and stress response [49]; circadian regulators *TOC1* and *ELF3*, which coordinate growth‒stress balance via light signaling [50] and are implicated in crop heterosis [51]; and *Hsp90.2-B*, which positively regulates photosynthetic efficiency and grain weight through actin-dependent chloroplast protein targeting [52].

### Allele-specific expression patterns in hybrid wheat

**Fig. 4:**
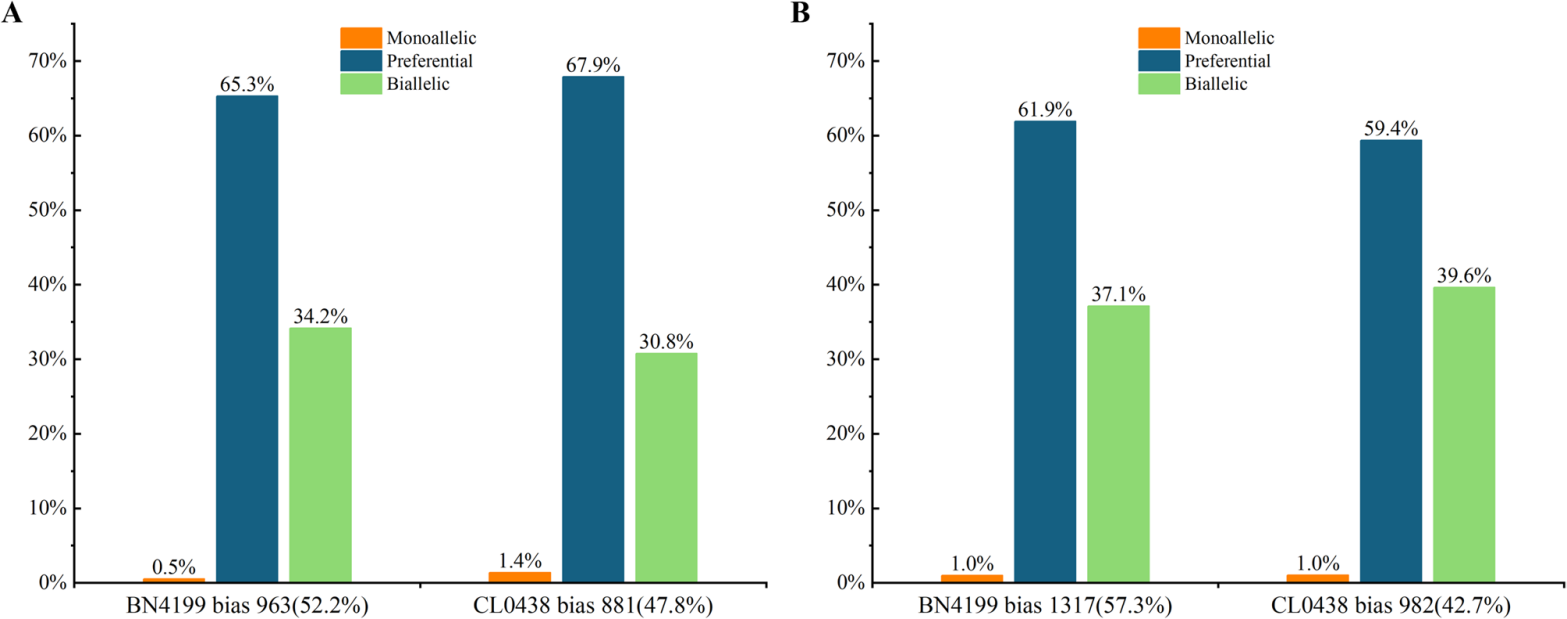
Allele-specific expression patterns in the hybrids at the anthesis (AN) and grain-filling (GF) stages. Proportions of genes with monoallelic expression, preferential expression, and biallelic expression profiles in the hybrids at the AN stage (**A**) and GF stage (**B**)

Allelic variation is widespread in plant genomes. Significant transcriptional divergence can occur between parental alleles of hybrids, representing one of the key molecular mechanisms underlying heterosis [​15]. The ASE analysis of the hybrid BC98 identified 31,862 SNPs at the AN stage and 20,048 SNPs at the GF stage in the transcribed regions of the genome, which corresponded to 5,982 (62.8% of total SNP-qualified genes) and 4,958 (64.0% of total SNP-qualified genes) protein-coding genes, respectively (Table 2). After correcting for Type I errors (*q* < 0.05), 1,844 ASE genes (30.8%) were detected at the AN stage (963 BN4199_BC98_-biased, 52.2% and 881 CL0438_BC98_-biased genes, 47.8%), which increased to 2,299 (46.4%) at the GF stage (1,317 BN4199_BC98_-biased genes, 57.3% and 982 CL0438_BC98_-biased genes, 42.7%) (Fig. 4A, Band Tables S10, S11). By classifying according to allelic expression ratios, we obtained the following results: monoallelic expression: 0.9% (AN) vs. 1.0% (GF); preferential expression: 66.5% (AN) vs. 60.8% (GF); and biallelic expression : 32.5% (AN) vs. 38.2% (GF). These ASE patterns revealed several key developmental dynamics in hybrid wheat. The increased predominance of BN4199-derived allelic bias at the GF stage suggests temporal activation of critical alleles from this parent, potentially regulating grain-filling or storage protein synthesis. This allelic bias highlights the dynamic selection of advantageous parental alleles across developmental phases. Furthermore, the observed increase in ASE genes from 1,844 at the AN stage to 2,299 at the GF stage indicates the progressive expansion of allele-specific regulatory networks in the hybrids, likely integrating complementary parental genetic effects to coordinate complex trait optimization during reproduction. Finally, the concurrent decline in preferential expression and the rise in balanced biallelic expression imply a developmental shift in regulatory strategies—from partial dominance driven by single-allele superiority to overdominance/dominance complementarity. These regulatory changes reinforce heterosis phenotypes through stage-specific modulation of allelic contributions.

**Table 2:** SNPs associated with allele-specific expression in hybrids across developmental stages

Developmental stages	SNPs for ASE analysis	Reads covered SNPs	Gene numbers	SNPs/Gene	Percentage of protein-coding genes
AN-stage	31,862	976,324	5,982	5.3	62.8%
GF-stage	20,048	977,679	4,958	4.0	64.0%

The developmental stage comparison of 2,988 ASE genes revealed 2,038 (68.2%) genes with stable allelic bias, 241 genes with switched preferences (132 switching from BN4199_BC98_- to CL0438_BC98_-biased alleles, 109 switching from CL0438_BC98_- to BN4199_BC98_-biased alleles), and 709 newly biased genes (Table 3). This plasticity is consistent with the ability of wheat to adaptively rewire its transcriptional processes during development [21]. The increased BN4199_BC98_ bias observed at the GF stage highlights the genome dominance of the parental line BN4199 during late-stage heterosis, which is consistent with prior studies that indicated regulatory divergence among the alleles in hybrids [15].

**Table 3:** ​Stage-specific analysis of allelic expression variation in the hybrids

		GF stage	
AN stage	BN4199_BC98_ bias	CL0438_BC98_ bias	No bias
BN4199_BC98_ bias	361	11	121
CL0438_BC98_ bias	7	204	102
No bias	391	318	1,473

The KEGG divergence underscores genes revealed distinct stage-specific metabolic priorities (Figure S3). At the AN stage, top enriched pathways included thiamine metabolism (ko00730), citrate cycle (TCA cycle) (ko00020), porphyrin and chlorophyll metabolism (ko00860), plant hormone signal transduction (ko04075), and alpha-Linolenic acid metabolism (ko00592), indicating emphasis on energy production and photosynthetic machinery. In contrast, at the GF stage, ASE genes prioritized glyoxylate and dicarboxylate metabolism (ko00630), glycine, serine and threonine metabolism (ko00260), plant hormone signal transduction (ko04075), protein processing in endoplasmic reticulum (ko04141), and TCA cycle (ko00020), highlighting shifts toward amino acid metabolism, protein modification, and carbon remobilization. Notably, plant hormone signal transduction (ko04075) and TCA cycle (ko00020) were conserved across stages.

### Developmental dynamics of *cis*- and *trans*-regulatory divergence

**Fig. 5:**
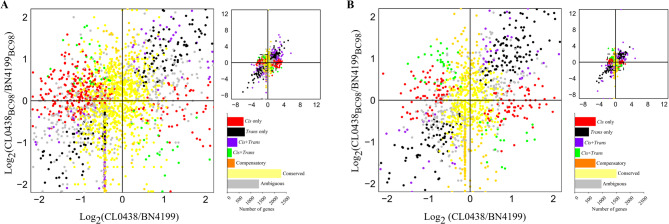
Classification of *cis- and trans-*regulation in wheat hybrids. The composite plot of the scatter plot and bar graph summarizes allele-specific expression divergence between the parental lines and the F_1_ hybrids. The genes ​are color-coded according to the classified *cis*- and *trans*-regulatory mechanisms. The bar graphs show the stage-specific gene counts of the regulatory categories detected in the wheat flag leaves at the anthesis (**A**) and grain-filling (**B**) stages

The regulatory divergence in the hybrid BC98 was dissected by comparing the ASE ratios (*cis* effects) and parental expression divergence (*trans* effects = total divergence − *cis* effects) across the developmental stages (Fig. 5A, B). Among the 5,982 genes at the AN stage, 11.5% (688 genes) exhibited *cis*-only regulation, 12.4% (742 genes) had *trans*-only regulation, and 15.5% (929 genes) had *cis-trans* interactions, whereas 38.2% (2,283 genes) exhibited no divergence (classified as “conserved”) (Tables S12, S13). Further examination of the 929 *cis*-*trans* interaction genes revealed three subtypes [53, 54]. In the enhancing subtype (*cis* + *trans*), both *cis*- and *trans*-regulatory mechanisms promoted the expression of identical parental alleles (423 genes, 7.1%). This subtype included identical and opposite scenarios (Table S12). In the antagonistic subtype (*cis* × *trans*), the *cis*- and *trans*-regulatory mechanisms acted in opposite directions on the same and different alleles (192 genes, 3.2%). In the compensatory subtype, *cis*-enhancement and *trans*-suppression of specific alleles offset each other, resulting in nonsignificant differences in gene expression between the parents or the hybrids, whereas the expression of the homologous allele remains​​ independently regulated (314 genes, 5.1%). At the GF stage, *cis*-only regulation increased to 15.2% (753/4,957 genes), surpassing *trans*-only regulation (10.2%, 508 genes) and compensatory regulation (15.0%, 744 genes) (Tables S12, S14). The cross-stage analysis of 7,736 genes revealed conserved regulatory patterns in 21.8% of the *cis*-only regulated genes, 9.4% of the *trans*-only regulated genes, and 30.7% of the *cis*-*trans* interaction genes (Table S15).

Developmental shifts were evident by the enhanced *cis*-only regulation at the GF stage (30.2% of the significant-effect genes vs. 29.2% at the AN stage), which agreed with the reproductive-phase *cis*-predominance observed in maize [16]. Moreover, *trans*-only regulation declined in the wheat hybrids (20.4% at the GF stage vs. 31.5% at the AN stage) (Table S12). Compensatory regulation increased from 5.1% at the AN stage to 15.0% at the GF stage, suggesting that adaptive balancing mechanisms were activated. Notably, the *cis*-*trans* regulatory genes at the GF stage presented a 1.3-fold expression bias toward the BN4199_BC98_ allele, which was also consistent with what was observed among the global expression trends.

### Associations between *cis*- and *trans*-regulatory divergence and gene expression patterns

**Fig. 6:**
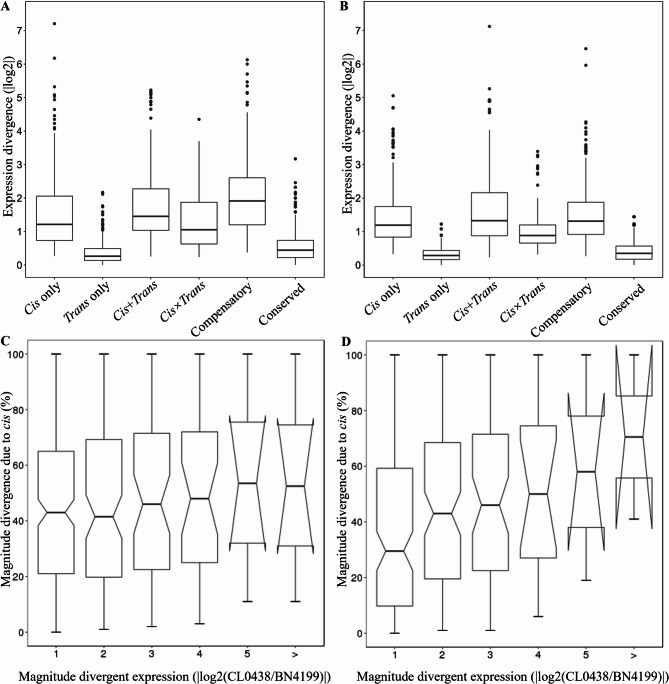
Contributions of cis- and trans-regulation at different developmental stages. The absolute magnitude of the gene expression divergence in the parental lines resulting from *cis*-only, *trans*-only, *cis* + *trans*, *cis* × *trans*, compensatory, and conserved regulation in wheat flag leaves at the anthesis (AN) stage (**A**) and grain-filling (GF) stage (**B**). Box-and-whisker plots showing the percent contribution of *cis*-only regulation (% *cis*) to total divergence, categorized by the magnitude of parental log_2_ expression differences (x-axis) in wheat flag leaves at the AN (**C**) and GF stages (**D**)

In hybrid plants, expression divergence between parental alleles may arise from variations in *cis*-regulation and/or *trans*-regulation [55]. This study compared the contributions of these regulatory mechanisms to expression divergence at different developmental stages. At the AN stage, the median absolute expression divergence attributable to *cis*-only (1.25-fold) and *trans*-only regulation (1.34-fold) was not significantly different (Wilcoxon test, *p* = 5.76e − 01) (Fig. 6A). In contrast, *cis*-only derived divergence significantly exceeded *trans*-only derived divergence at the GF stage (1.32-fold vs. 1.13-fold; *p* < 2.34e − 05) (Fig. 6B), with the *cis*-only contribution (% *cis*) increasing proportionally to the magnitude of the divergence [16] (Fig. 6C, D). Overall, the developmental transition from *trans*- to *cis*-dominant regulatory control likely reflects the biological shift of wheat from growth plasticity at the AN stage to metabolic specialization during the GF stage. This regulatory rewiring balances developmental flexibility (mediated by early *trans*-regulation) with trait stability (enforced by late *cis*-regulation), providing a mechanistic foundation for heterosis.

Non-additive gene expression was the most common form of gene expression at the AN stage, particularly within the *trans*-only (45.9% BN4199-dominant) and *cis*×*trans* antagonistic (46.2% BN4199-dominant) categories (Table S16). *Cis*-only genes presented relatively greater proportions of additive expression (45.3% at the AN stage, increasing to 55.4% at the GF stage). These findings indicate that *cis*-only regulation prefers additive expression at the GF stage, which likely stabilizes developmental processes by minimizing transcriptional noise and sensitivity to environmental cues. This phenomenon has also been observed in maize [16, 56]. *Cis*×*trans* antagonistic regulation had the lowest proportion of additive expression (34.6% at the AN stage, 44.4% at the GF stage) and was the primary driver of non-additive expression. Additionally, *cis*×*trans* antagonistic regulation primarily mediated positive overdominance in this study, ​​which is consistent​​ with previous findings that *cis*×*trans* antagonism promotes transgressive expression [57]. At the GF stage, *trans*-only and *cis*×*trans* antagonistic regulation exhibited significant biases toward BN4199 (33.0% and 30.6%, respectively), highlighting their importance in BN4199-dominant allele expression.

Collectively, these processes support a two-phase model, which states that transient *trans*-driven responses at the AN stage shift to stable *cis*-dominant regulation at the GF stage (Fig. 5A, B and Table S12). The rice HoliB model demonstrated that insufficient genetic backgrounds (e.g., low transcription factor/substrate availability) lead to functional constraints in parental homozygotes, thereby generating non-additive effects in F^1^ hybrids. Conversely, optimized backgrounds restore additive expression patterns by alleviating these limitations [45]. Our two-phase model extends the rice paradigm by integrating developmental stage-specific transitions in regulation.

### Identification of gene co-expression modules in the hybrid

Gene co-expression network analysis enables the identification of functionally coordinated gene modules and reveals how their expression patterns are associated across samples. This approach helps uncover potential biological heterogeneity. Using WGCNA, 23,719 expressed genes were found to be clustered into 18 co-expression modules (Figure S4), representing gene clusters with significantly coordinated expression patterns. We focused subsequent analysis on the Brown and Red modules based on three key criteria (Figure S5):​​ (1) ​​Stage-specific association at the target phase:​​ In the hybrid BC98 at the GF stage, the Brown module exhibited the highest positive ME value (ME = 0.20), while the Red module exhibited the most negative ME value (ME = −0.27); (2) ​​Largest magnitude of dynamic change:​​ The Red module displayed the greatest shift in ME value within hybrid BC98, changing from ME = 0.33 at the AN stage to ME=−0.27 at the GF stage (ΔME = 0.60), the largest ΔME observed among all modules; (3) ​​Significant Genotype × Developmental Stage interaction:​​ Multifactor ANOVA confirmed a highly significant interaction effect for both modules (Brown: *F* = 12.65, *p* < 0.001; Red: *F* = 8.96, *p* = 0.003), strongly linking their expression patterns to heterosis and developmental transition. This highlights the spatiotemporal regulatory role of these modules in heterosis. These findings also align with previous reports on dynamic co-expression module regulation during developmental transitions in wheat spikes [58], maize inflorescences [59], and rice flowering time [60]. Consequently, the Red and Brown modules were prioritized for subsequent analyses.

**Fig. 7:**
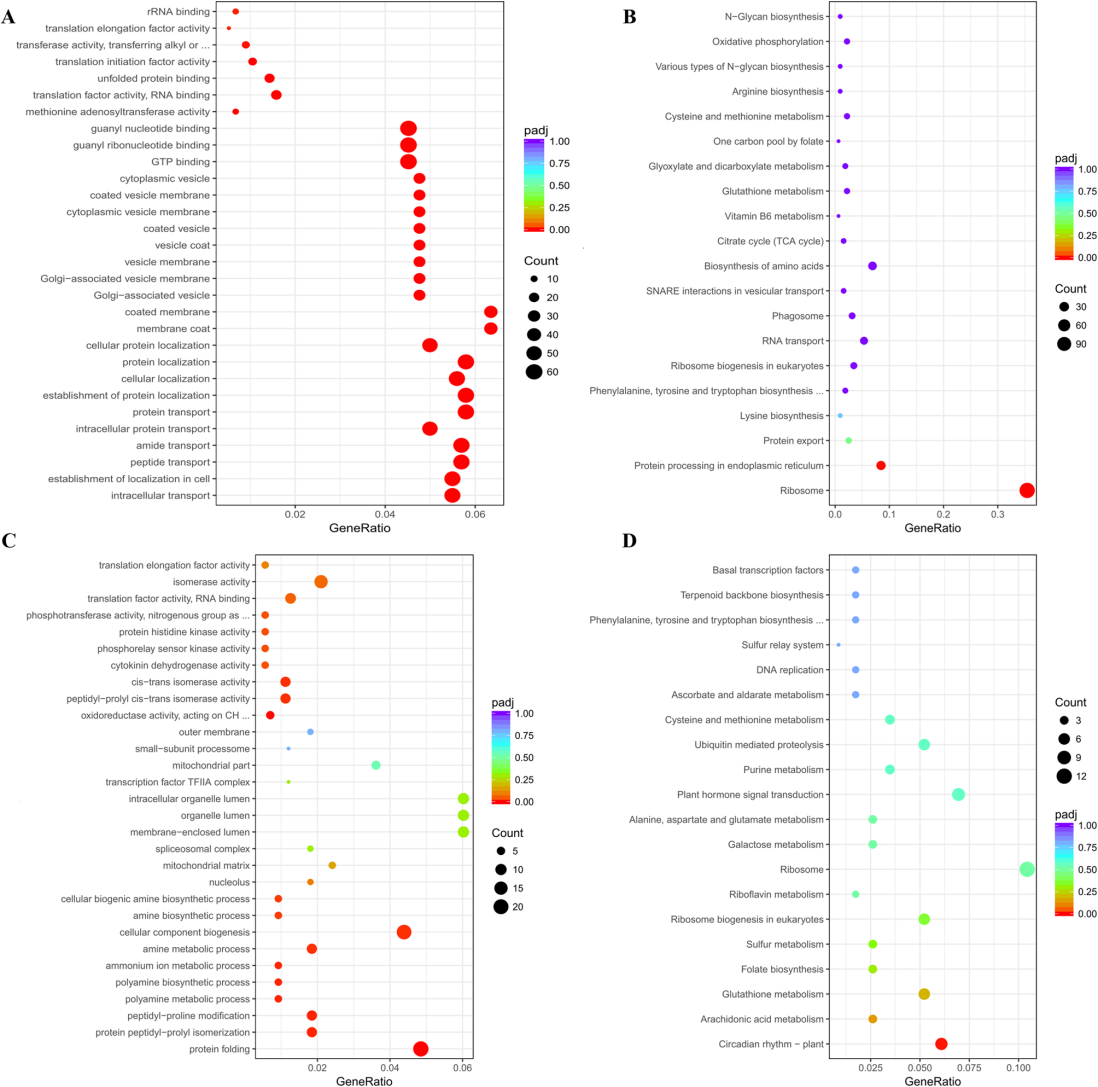
Functional enrichment and pathway analysis of co-expression modules. (**A**) GO term enrichment of the Brown module; (**B**) KEGG pathway enrichment bubble plot of the Brown module; (**C**) GO term enrichment of the Red module; (**D**) KEGG pathway enrichment bubble plot of the Red module

The ME value of the Brown module increased from the AN to GF stage (ΔME = + 0.47), accompanied by enrichment in three GO categories: BP associated with “protein transport localization”, CC associated with “vesicle membrane systems”, and MF associated with “GTPase activity” (Fig. 7A, Table S17). These findings collectively revealed a hierarchical regulatory network underlying heterosis formation when plants are transitioning between the AN stage and the GF stage, where GTPase activity (MF) drives vesicle transport (CC), which mediates protein targeting (BP). This coordinated mechanism markedly enhances protein transport efficiency during the GF stage. Its regulatory timing perfectly synchronizes with peak protein synthesis and translocation demands in flag leaves while transitioning to the reproductive phase. The KEGG pathway analysis further corroborated these results, identifying enriched pathways, such as “protein processing in endoplasmic reticulum” (ko04141) and “protein export” (ko03060), which together form a “synthesis-processing-transport” axis (Fig. 7B, Table S18). This network likely enhances photosynthate unloading through ER chaperones and Rab GTPases [61, 62], while its suppression at the AN stage allows pollen development to be prioritized [63]. The Red module displayed significant activation at the AN stage (*p* < 0.001) and was enriched for polyamine metabolism (GO:0006595/6), protein folding (GO:0006457), and mitochondrial/nucleolar components (Fig. 7C, Table S19). Its downregulation at the GF stage coincided with the beginning of leaf senescence [64], suggesting that the source‒sink transitions were mediated by a circadian rhythm (ko04712) through kinase/isomerase activity (Fig. 7D, Table S20) [65, 66].

### Identification of hub genes and transcription factors in the target modules

**Fig. 8:**
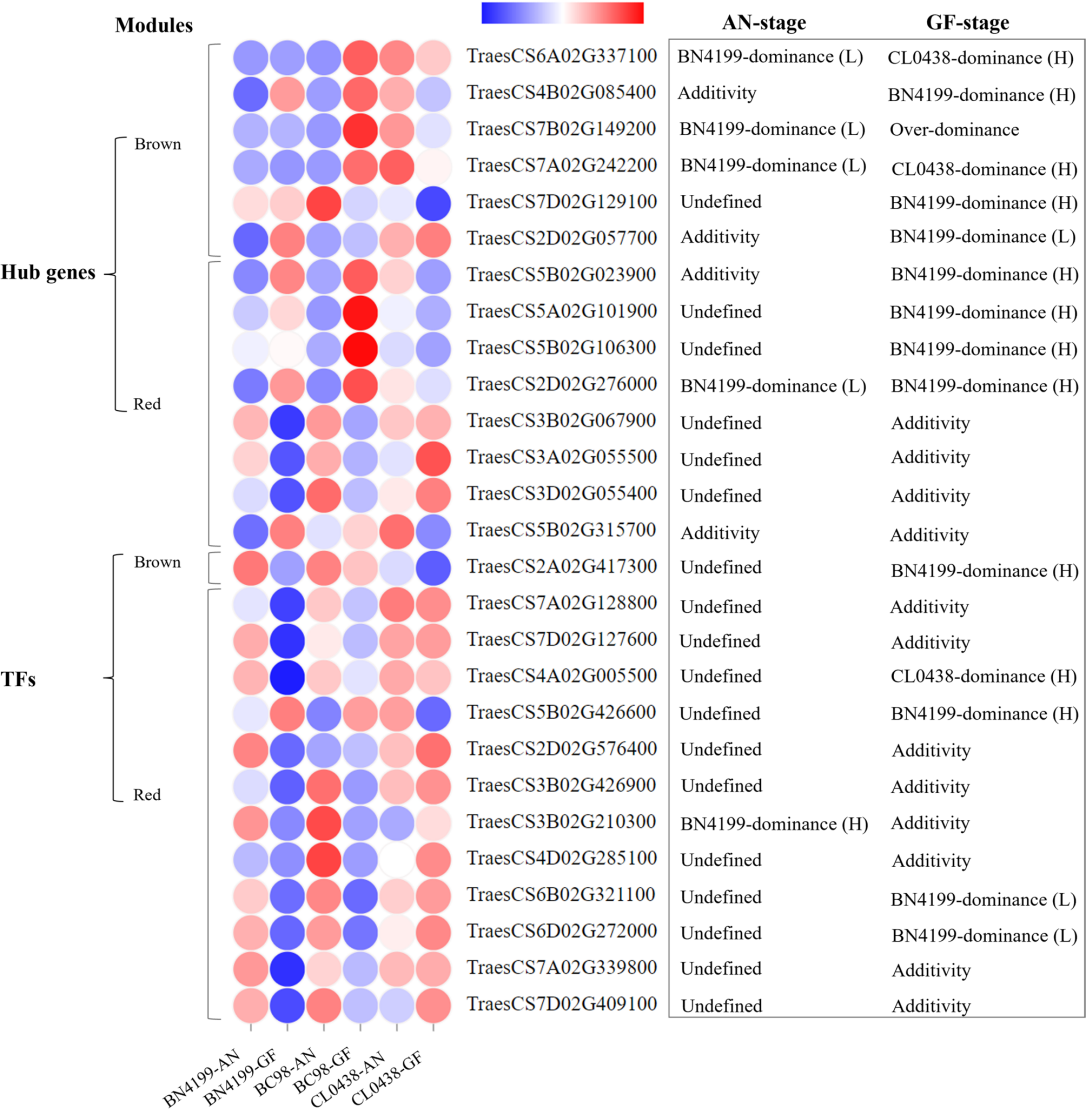
Hub genes and key transcription factors (TFs) identified in the two target modules. L and H between parentheses denote low-parent dominance and high-parent dominance, respectively. The AN and GF stages represent the anthesis and grain-filling stages, respectively. The color gradient from blue to red indicates increasing gene expression levels. The blue‒white‒red gradient represents Z score-normalized expression levels (µ = 0, σ = 1), with blue indicating baseline expression (Z = 0) and red denoting significant upregulation (Z ≥ 2)

Co-expression network analysis revealed 14 hub genes potentially associated with heterosis, including six genes from the Brown module and eight from the Red module (Fig. 8; Figure S6A, B; Tables S21-S23). In addition, 13 transcription factors (TFs) belong to the AP2/ERF, bHLH, HD-ZIP, MYB, NAC, and Dof families (Table S24). The Brown module included hub genes that regulate protein homeostasis (e.g., TraesCS2D02G057700 encoding a kinase/chaperone [67]) and stress adaptation (TraesCS7A02G242200 encoding an ATPase [68]). Notably, the hub gene *HSP90.2-B* (TraesCS7B02G149200) orchestrates various biological processes. For example, this gene orchestrates chloroplast precursor protein transport to increase photosynthetic efficiency and grain filling [52], gibberellin-mediated hormone signaling to optimize reproductive development [69], metabolic enzyme stabilization under abiotic stress [70], and molecular scaffolding to coordinate the developmental transition from vegetative to reproductive growth [71]. In this study, *HSP90.2-B* exhibited BN4199-biased expression at the AN stage and overdominance at the GF stage. These developmental stage-specific dominance dynamics and their functional versatility suggest that *HSP90.2-B* potentially serves as a key regulator of heterosis and associated yield-related traits in hybrid wheat. The Red module, which is activated at the AN stage, harbors hub genes that primarily govern stress-responsive isomerase activity (TraesCS2D02G276000) [72] and sugar metabolism (TraesCS3B02G067900) [73]. A key AP2/ERF family transcription factor (TF) in the brown module (TraesCS2A02G417300) exhibited BN4199-biased high-parent dominance at the GF stage (Fig. 8), suggesting its role in increasing yield in hybrid plants. Similarly, the AP2/ERF and NAC TFs in the red module coordinate the stress response and transitions between developmental stages [74, 75]. qRT‒PCR validation confirmed the reliability of the RNA-seq data (R² = 0.85) (Figure S7 and Table S25).

## Discussion

### The role of gene expression variation in hybrid systems

The relationship between parental genetic distance and heterosis remains controversial. Boeven et al. [76] reported that heterosis in elite wheat hybrid combinations increased with greater genetic distance, whereas other studies reported weak correlations [77], and some rice studies even reported negative correlations between yield-related traits and genetic distance [78]. These contradictions suggest that genetic distance alone is insufficient to predict heterosis and that moderate genetic differentiation is crucial for optimal hybrid performance. In this study, the high number of DEGs between BN4199 and CL0438 (68.5% at the AN stage and 46.7% at the GF stage) reflects their genetic divergence but remains within a range conducive to heterosis. Notably, the hybrid BC98 exhibited a parental BN4199-biased transcriptional pattern. The number of DEGs between BC98 and BN4199 (5,623 at the AN stage; 3,218 at the GF stage) was significantly lower than that between BC98 and CL0438 (9,108 at the AN stage; 4,562 at the GF stage). Hierarchical clustering further confirmed this trend (Fig. 2D). Phenotypically, BN4199 contributes key traits such as thousand-grain weight and grain yield per plant. Moreover, CL0438 presented complementary characteristics, revealing a synergistic mechanism whereby parental alleles collectively enhance heterosis. Together, these findings demonstrate that moderate genetic divergence, coupled with transcriptional dominance from the superior parent and phenotypic complementarity, synergistically drives the robust heterosis observed in BC98.

### Dynamic shifts between additive and non-additive expression patterns underpin heterosis

The debate over whether heterosis is driven by additive or non-additive gene expression has been extensively discussed. For tobacco, Duan et al. [79] reported that dominant and overdominant DEGs collectively accounted for more than 60% of the expression changes in hybrid leaves. In maize, studies on hybrid crosses revealed that most DEGs presented non-additive expression patterns [59]. However, conflicting maize studies argue that additive expression predominantly drives heterosis [16, 59]. In rice, emerging evidence indicates that yield-related heterosis is regulated primarily by non-additive gene effects [45, 80], whereas opposing studies report additive expression as the dominant pattern in hybrids [81]. Synthetic hexaploid wheat studies suggest that high parental expression-level dominance of protein-coding genes is correlated with growth vigor [82]. These findings indicate that heterosis likely arises from species- or stage-specific regulatory mechanisms balancing additive and non-additive effects rather than from a universal model. In this study, transcriptome dynamics in the wheat hybrid BC98 revealed stage-specific regulatory shifts: non-additive expression dominated at the AN stage (60.5% of DEGs), whereas additive patterns increased significantly by the GF stage (49.3% of DEGs) (Fig. 3A and Table S3), suggesting temporal coordination between genetic mechanisms. Notably, non-additive genes decreased sharply (47.9% reduction) from the AN stage to the GF stage, reflecting their developmental plasticity, whereas additive genes exhibited greater stability (21.8% reduction), aligning with their role in maintaining basal metabolism and homeostasis. These findings support a “dual-engine” model: early growth vigor is driven by dynamic non-additive interactions, whereas late-stage grain filling relies on additive complementation to stabilize yield-related traits.

The conservation and transition of expression patterns reflect developmental priorities. The observed stability in non-additive expression patterns (30.1% conserved) likely stems from the sustained activation of key regulatory networks established during the AN stage. Genes maintaining non-additivity may be governed by stable epigenetic modifications or consistently expressed regulatory factors in the hybrid context, ensuring the continuation of advantageous traits such as stress protection or growth vigor signaling pathways. Notably, the significant shift toward additive patterns (20.3% transitioned) aligns with the increasing demand for metabolic precision and homeostasis during the GF stage. Our KEGG analysis of additive genes provides critical mechanistic insights into this transition. The enrichment of core metabolic pathways (e.g., starch and sucrose metabolism, phenylpropanoid biosynthesis) at both stages underscores their fundamental role in cellular maintenance, executed through additive expression. More importantly, the developmental stage-specific reprogramming from pathways supporting active defense implementation (e.g., plant-pathogen interaction, diterpenoid biosynthesis) at the AN stage to pathways prioritizing reproductive resource allocation (e.g., flavonoid biosynthesis, plant hormone signaling, ABC transporters) at the GF stage demonstrates how additive expression facilitates environmental adaptability. It is worth noting that the Brown module, significantly enriched at the GF stage and functionally linked to vesicle transport and endoplasmic reticulum protein processing, may directly contribute to stabilizing this additive expression landscape. By enhancing the efficiency of protein synthesis, processing, and targeted transport (e.g., photoassimilate unloading), this module ensures the reliable execution of additively controlled metabolic and signaling pathways essential for grain-filling stability. Together, these findings provide a molecular foundation for the temporal transition of heterosis from early plasticity to late-stage yield stability.

Functional enrichment analysis further elucidated this dichotomy. Additive genes were consistently enriched in core metabolic processes (e.g., primary metabolism, catalytic activity) across both stages, underscoring their foundational role in cellular maintenance. In contrast, BN4199-dominant non-additive genes presented stage-specific specialization, with metabolic pathway activation at the AN stage and stress-response and developmental regulation at the GF stage. Our KEGG analysis further elucidates how BN4199-dominant alleles orchestrate metabolic hierarchies to enhance heterosis. These genes occupy pivotal nodes in carbon and protein homeostasis networks, such as ​​*RBCS*​​ and *​​SPS*​​ in photosynthetic carbon flow, and *​​HSP90.2-B*​​ in chaperone-mediated protein folding. This enables efficient resource channeling toward grain development. The dominance of BN4199 alleles in rate-limiting enzymes (e.g., ​​*SPS*​​) and upstream regulators (e.g., AP2/ERF TFs) suggests a multi-layered control. Transcriptional activation of metabolic genes establishes a “source” strength in flag leaves, while protein stabilization mechanisms ensure “sink” capacity in grains. This hierarchical optimization likely underlies the robust yield heterosis observed in BC98, where BN4199’s genetic repertoire acts as a master regulator of physiological efficiency. Through functional enrichment analysis and identification of key genes such as *RBCS* involved in photosynthesis and *HSP90.2-B* regulating chloroplast protein targeting, this study reveals that BN4199 enhances hybrid vigor via stage-specific metabolic and regulatory networks during the AN and GF stages. The interaction between stable additive and plastic non-additive effects, regulated by genetic background and epigenetic mechanisms, demonstrates how heterosis arises through stage-specific optimization of parental allele expression patterns.

### Developmental stage-specific dynamics of allele-specific expression

Allelic variation, which is prevalent in plant genomes, generates diverse gene expression patterns through hybridization, thereby influencing phenotypic traits [83]. In this study, the percentage of genes exhibiting ASE increased from 30.8% at the AN stage to 46.4% at the GF stage, with a concurrent increase in parental BN4199 bias (52.2–57.3%) (Fig. 4 and Tables S10, S11). These results highlight the developmental stage dependency of ASE patterns. Notably, the ASE status of approximately 32% of the genes changed during the AN-to-GF transition (Table 3), reflecting transcriptional plasticity, which likely adapts to shifting physiological demands from reproductive growth to grain filling. Similar regulatory plasticity has been reported in wheat developmental studies [21].

The KEGG divergence underscores developmental reprogramming of heterosis mechanisms. AN-stage ASE genes were enriched in photosynthesis-associated pathways (Porphyrin and chlorophyll metabolism (ko00860)) and energy-generating processes (TCA cycle (ko00020)), aligning with early growth vigor demands for pollen development and biomass accumulation. Conversely, GF-stage ASE genes dominated amino acid metabolism and endoplasmic reticulum protein processing, consistent with sink-strengthening roles in nitrogen assimilation and storage protein synthesis. The persistent enrichment of plant hormone signaling and TCA cycle suggests core regulatory roles in stage transitions, while the emergence of glyoxylate metabolism, a key photorespiration bypass pathway, reflects optimized carbon recovery during the GF stage. This metabolic shift from photosynthetic dominance at the AN stage to nutrient reallocation at the GF stage demonstrates how allele-specific expression precisely regulates heterosis formation according to the physiological demands at different developmental stages in wheat.

### *Cis*- and *trans-* regulation drives stage-specific changes in gene expression patterns

The interplay between *cis*- and *trans*-regulatory divergence exhibited distinct developmental stage specificity, reflecting a coordinated shift in the transcriptional control underlying heterosis. At the AN stage, *trans-*only regulation dominated (31.5% of significant-effect genes), facilitating rapid, non-additive expression patterns critical for early growth vigor. In contrast, the GF stage presented a marked increase in *cis*-only regulation (30.2% of significant-effect genes), aligning with the stabilization of additive expression to ensure resource allocation and yield stability. Notably, *cis*×*trans* antagonistic regulation emerged as a key driver of overdominant expression, enabling transgressive allele interactions. This regulatory plasticity mirrors findings in maize, where *cis*-regulation gains prominence during reproductive phases to buffer environmental noise [16, 56]. The shift from *trans*- to *cis*-dominant control supports a two-phase model: *trans*-driven dynamic responses during the AN stage transition to *cis-*mediated stability in the GF stage, balancing developmental flexibility with trait optimization. These dynamics, coupled with the persistent BN4199 allelic bias in *trans*-only and *cis*×*trans* regulation, underscore the superior parent’s role in harmonizing heterotic outcomes through stage-specific regulatory rewiring. While the rice HoliB model established that background insufficiency underlies non-additive effects, while background optimization shifts expression toward additive patterns [45], our findings extend this understanding. We revealed that in wheat, the precise temporal coordination of *cis*- and *trans*-regulatory dynamics, coupled with the preferential expression of the superior parent BN4199 alleles, collectively optimizes the gene regulatory network across developmental stages. This discovery advances the existing theory and provides novel mechanistic insights into heterosis in wheat.

### Systematic comparison of heterosis regulation in wheat, maize, and rice

While this study reveals stage-specific regulatory dynamics underlying wheat heterosis, comparisons with maize and rice highlight conserved and divergent mechanisms across cereals. Similar to maize, wheat exhibits a developmental shift toward *cis*-regulatory dominance and additive expression patterns during reproductive stages such as grain-filling, which stabilizes yield-related traits [16, 56]. Rice heterosis often favors non-additive expression, particularly in indica-japonica hybrids and early development; conversely, the transition from non-additive to additive expression in wheat appears more pronounced than that documented in rice [45, 80].

However, wheat displays unique regulatory features. First, unlike maize and rice, wheat hybrids show a progressive increase in superior-parent (BN4199) allelic bias during development, rising from 52.2% at the AN stage to 57.3% at the GF stage. This positions the superior parent as a developmental-stage-modulated allelic reservoir. Second, wheat demonstrates distinctive stage-specific partitioning between non-additive and additive expression. Its dual-engine model features non-additive dominance at the AN stage (60.5% of DEGs) shifting to additive enrichment at the GF stage (49.3%), exceeding the temporal plasticity observed in maize and rice [16, 81], where additive effects dominate more uniformly across stages. Third, we identified that wheat hybrids utilize *cis* × *trans* antagonistic interactions as the primary driver of overdominant expression, a mechanism not reported in either rice or maize hybrids. Interestingly, in parental maize lines, *cis* and *trans* synergistic interactions within each parent have been shown to enable gene-specific hyperexpression [84], revealing fundamentally different regulatory architectures between these cereal species. These distinctions likely arise from the polyploid nature of wheat, which may amplify developmental-stage-specific tuning of parental contributions. Nevertheless, the conserved enhancement of expression levels driven by *cis*-*trans* interactions underscores a unified evolutionary strategy for hybrid vigor across cereal species.

### Stage-specific regulatory networks underlie heterosis mechanisms

The functional enrichment of co-expression modules revealed temporally distinct regulatory networks driving heterosis. The Brown module, which was upregulated during the GF stage, was enriched for protein transport, vesicle trafficking, and GTPase activity. This finding suggests its role in optimizing resource allocation by enhancing photosynthate unloading to grains. Conversely, active at the AN stage, the Red module links polyamine metabolism and circadian rhythm pathways, potentially coordinating early growth vigor through energy-metabolic reprogramming. Notably, hub genes such as the *HSP90.2-B* gene in the Brown module exhibited stage-specific dominance, transitioning from BN4199-biased expression at AN to overdominance at the GF stage, aligning with its multifunctional roles in chloroplast protein trafficking and stress adaptation [52, 69–71]. Similarly, the Red module’s AP2/ERF and NAC transcription factors modulate developmental transitions and stress responses, reflecting their regulatory versatility [74, 75]. These modules collectively orchestrated a hierarchical network: Brown-mediated protein homeostasis stabilized yield traits during the GF stage, whereas red-driven metabolic flexibility supported dynamic growth at the AN stage. The spatiotemporal specialization of these modules highlights how heterosis emerges from developmental stage-specific optimization of parental genetic contributions, integrating metabolic efficiency, stress resilience, and resource partitioning.

## Conclusion

**Fig. 9:**
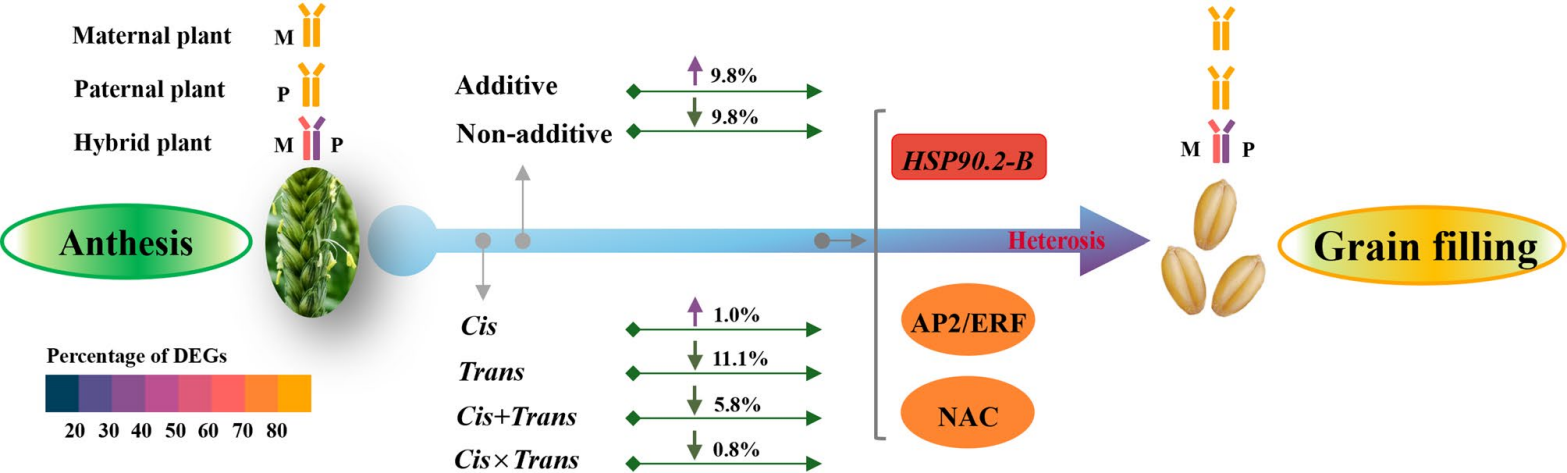
Schematic diagram of the stage-specific molecular regulatory network underlying heterosis in flag leaves of hybrid wheat. The gradient color blocks represent the percentage of differentially expressed genes with maternal (M) BN4199 dominance and paternal (P) CL0438 dominance among non-additive genes in the hybrid plant at the anthesis (AN) and grain-filling (GF) stages. The gradient-colored arrowed horizontal axis and the green rightward long arrow represent the developmental transition from the AN stage to the GF stage. Purple upward arrows indicate an increasing proportion of genes under this pattern, while green downward arrows indicate a decreasing proportion of genes

This study explored the molecular mechanisms underlying heterosis in the hybrid wheat line BC98 by analyzing its transcriptional dynamics at the AN and GF stages (Fig. 9). The results revealed that the hybrid plants presented a systemic allelic preference for the superior parent BN4199. As the primary donor of favorable alleles, BN4199 is responsible for various beneficial pathways, such as carbon metabolism and stress response, which collectively improve the hybrid’s photosynthetic efficiency and grain development. We also observed that the allele-specific regulatory network in hybrids gradually expanded during development, with hybrids dynamically selecting advantageous alleles at different developmental stages. Some key advantageous parental alleles are selectively activated in later developmental stages. The hybrid also displays a developmental stage-dependent gene expression pattern and a temporally specific functional division between *cis*- and *trans*-regulation. Non-additive genes dominate the regulatory network at the AN stage, which drives early growth vigor. In contrast, additive genes become significantly enriched at the GF stage and contribute to stabilizing grain development and yield-related traits. The preferential association of *cis*-regulation with additive gene expression and *trans*-regulation with non-additive gene expression explains the high correlation between these two regulatory layers. These two mechanisms support the “dual-engine” model of heterosis, which consists of dynamic non-additive regulation and systemic additive compensation. This model states that the synergistic interactions that occur between the developmental stages and the plants’ genetic background produce phenotypic advantages in a spatiotemporal manner. The developmental stage-specific division in *cis*- and *trans*-regulation can also be explained via a two-phase model in which wheat transitions from *trans*-driven early growth vigor to *cis*-mediated grain filling. Notably, *cis*×*trans* interactions consistently serve as the core drivers of overdominance at both stages. We propose that the allelic superiority of BN4199 arises from long-term selection shaping functional pleiotropy, centrality in regulatory networks, stability of *cis*-regulatory elements, and dominant/overdominant effects. These attributes are amplified in hybrids via stage-specific expression patterns, ultimately driving high and stable yield heterosis. Additionally, WGCNA identified two key modules: the Brown module, which is associated with vesicle transport at the GF stage, and the Red module, which is involved in polyamine-circadian rhythm coordination at the AN stage. The regulatory network also shapes hub genes (e.g., *HSP90.2-B*) and transcription factors (e.g., AP2/ERF, NAC). Nevertheless, the broader applicability of these findings should be interpreted considering methodological factors. While the hybrid combination investigated represents a regionally typical heterosis pattern, the generalizability of the models requires further validation using additional hybrid combinations exhibiting strong heterosis. Additionally, the complex regulatory networks in hexaploid wheat, involving subgenome interactions, increase analytical complexity. Although a unique mapping strategy was employed to minimize interference from homoeologous genes, this study could not fully resolve the intricate mechanism of allele-specific effects potentially involving subgenome interactions in hexaploid wheat. Collectively, these findings characterize the multi-layered cooperative networks regulating heterosis development, advancing our understanding of the molecular mechanisms underlying wheat hybrid vigor.

## Supplementary Information

Supplementary Material 1.

Supplementary Material 2.

## Data Availability

The datasets supporting the findings of this study are included within the article and its supplementary materials. The raw Illumina sequencing data have been deposited in the NCBI Sequence Read Archive under accession number SRP585076 (https://www.ncbi.nlm.nih.gov/sra/%20SRP585076).
