# Supplementary material for: Transcriptome dynamics and allele-specific regulation underlie wheat heterosis at the anthesis and grain-filling stages
Source: BMC Genomics. 2025 Sep 2;26:798. doi: 10.1186/s12864-025-11983-2 (PMC12403350; doi:10.1186/s12864-025-11983-2)
Supplement: Supplementary file 1 — Supplementary Material 1. [file 12864_2025_11983_MOESM1_ESM.docx]

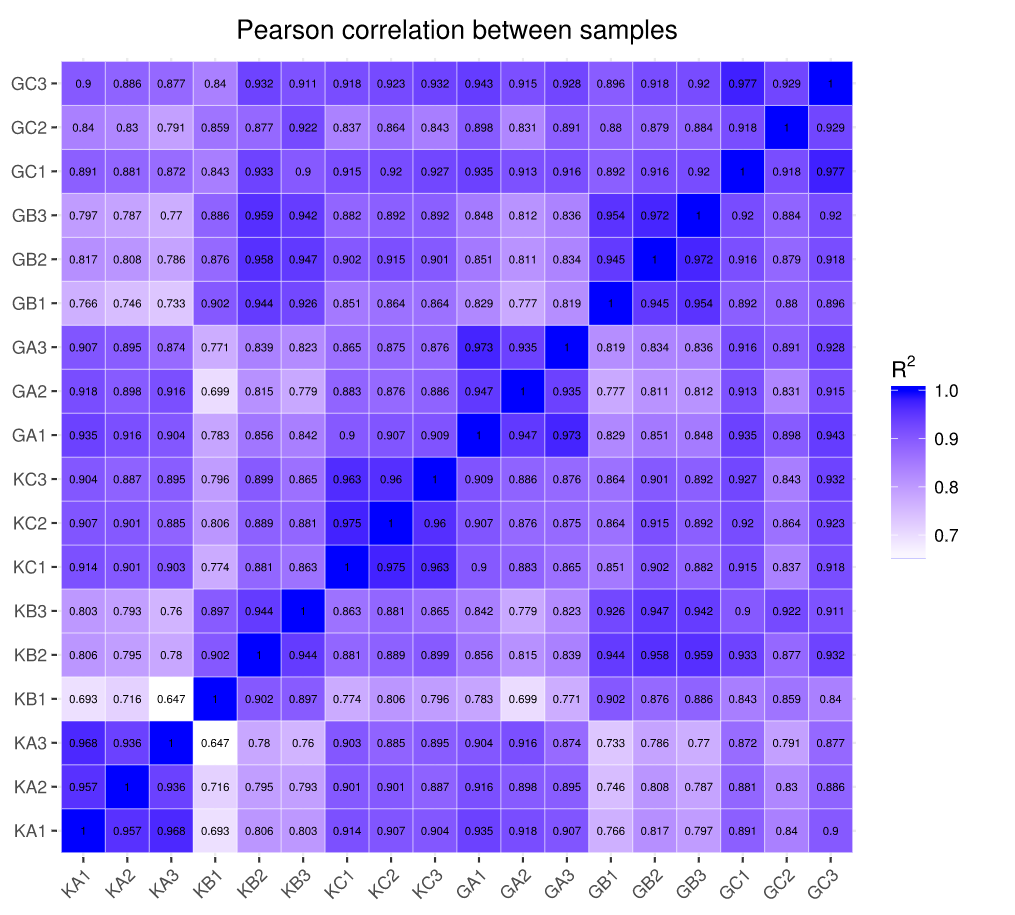


**Figure S1** Pearson’s correlation coefficient among three biological replicates of each genotype. KA, KB, and KC indicate BN4199, CL0438, and BC98 at the anthesis stage; GA, GB, and GC represent these genotypes at the grain-filling stage; numbers 1, 2, and 3 indicate the three biological replicates.


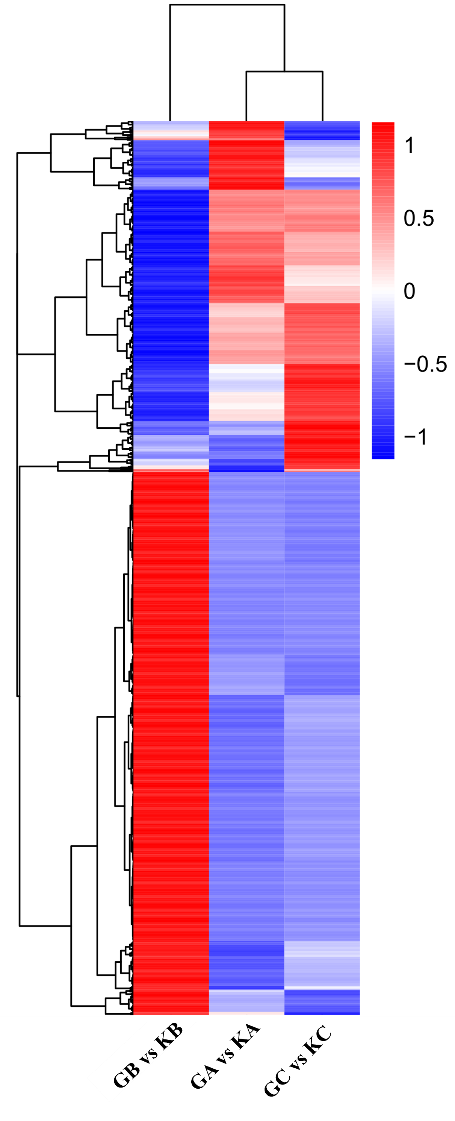


**Figure S2** Hierarchical cluster analysis of the 1,088 differentially expressed genes. The color key represents log_2_ (fold change). KA, KB, and KC indicate BN4199, CL0438, and BC98 at the anthesis stage; GA, GB, and GC represent these genotypes at the grain-filling stage. The color scale represents log_10_(FPKM +1) values, with red indicating relatively high expression and blue indicating relatively low expression.


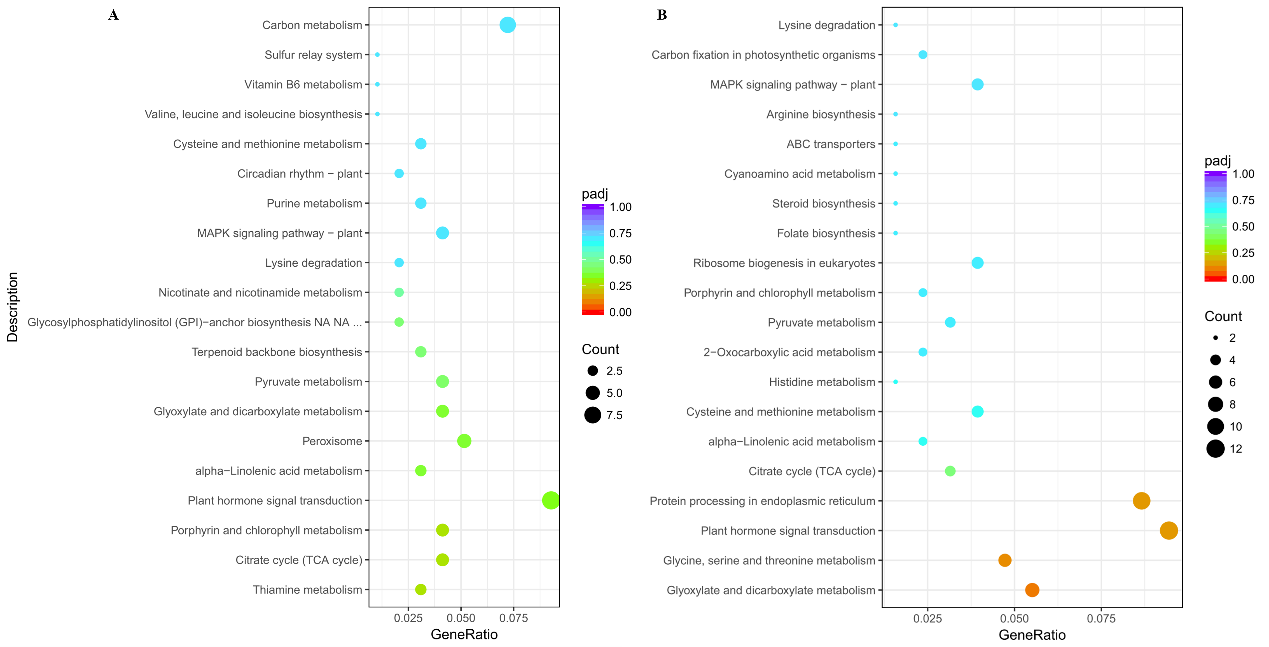


**Figure S3** Developmental stage-dependent KEGG enrichment of ASE genes​​. (A) Anthesis-stage top 20 pathways. (B) Grain-filling-stage top 20 pathways.


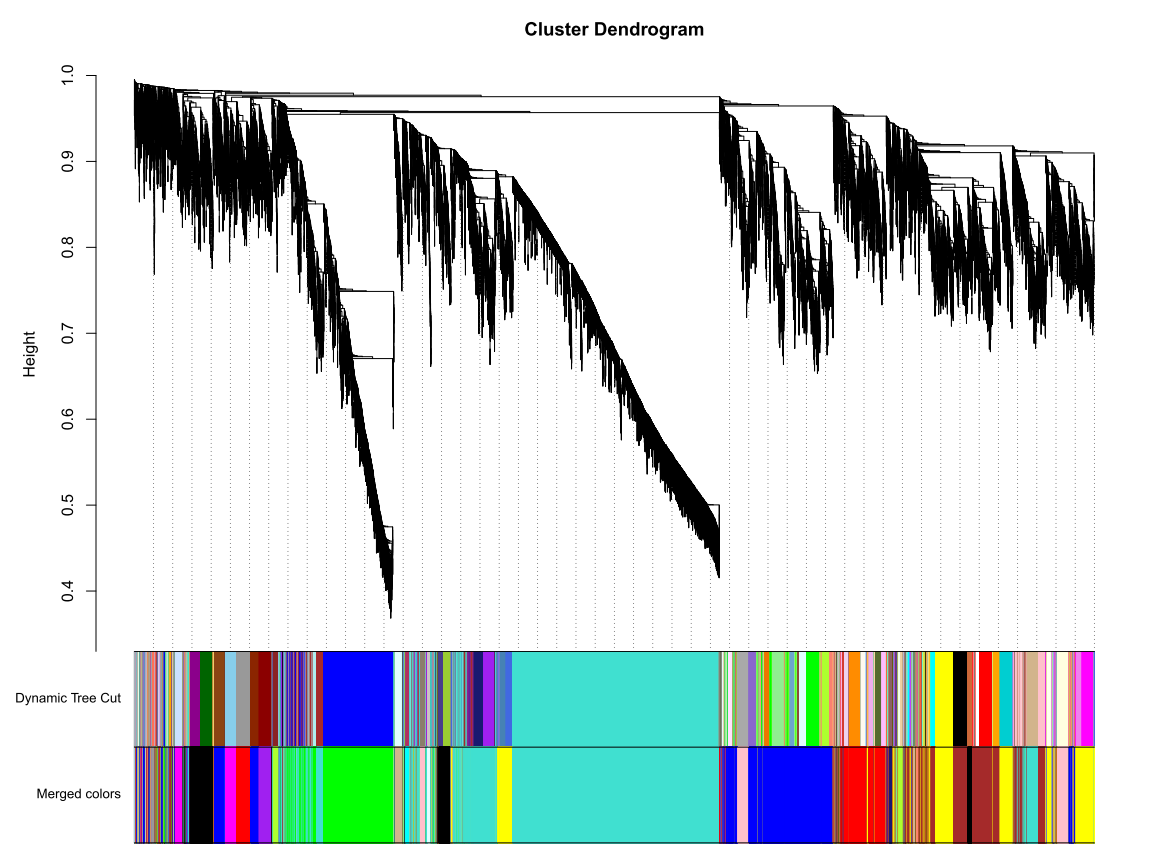


**Figure S4** Identification of co-expression modules through hierarchical clustering. The dendrogram was generated using pairwise gene correlation coefficients, with distinct branches corresponding to individual modules. Module identification was performed using the Dynamic Tree Cut algorithm, followed by merging closely related clusters to form consolidated modules, as denoted by merged color labels. These finalized modules were utilized for subsequent functional and network analyses.


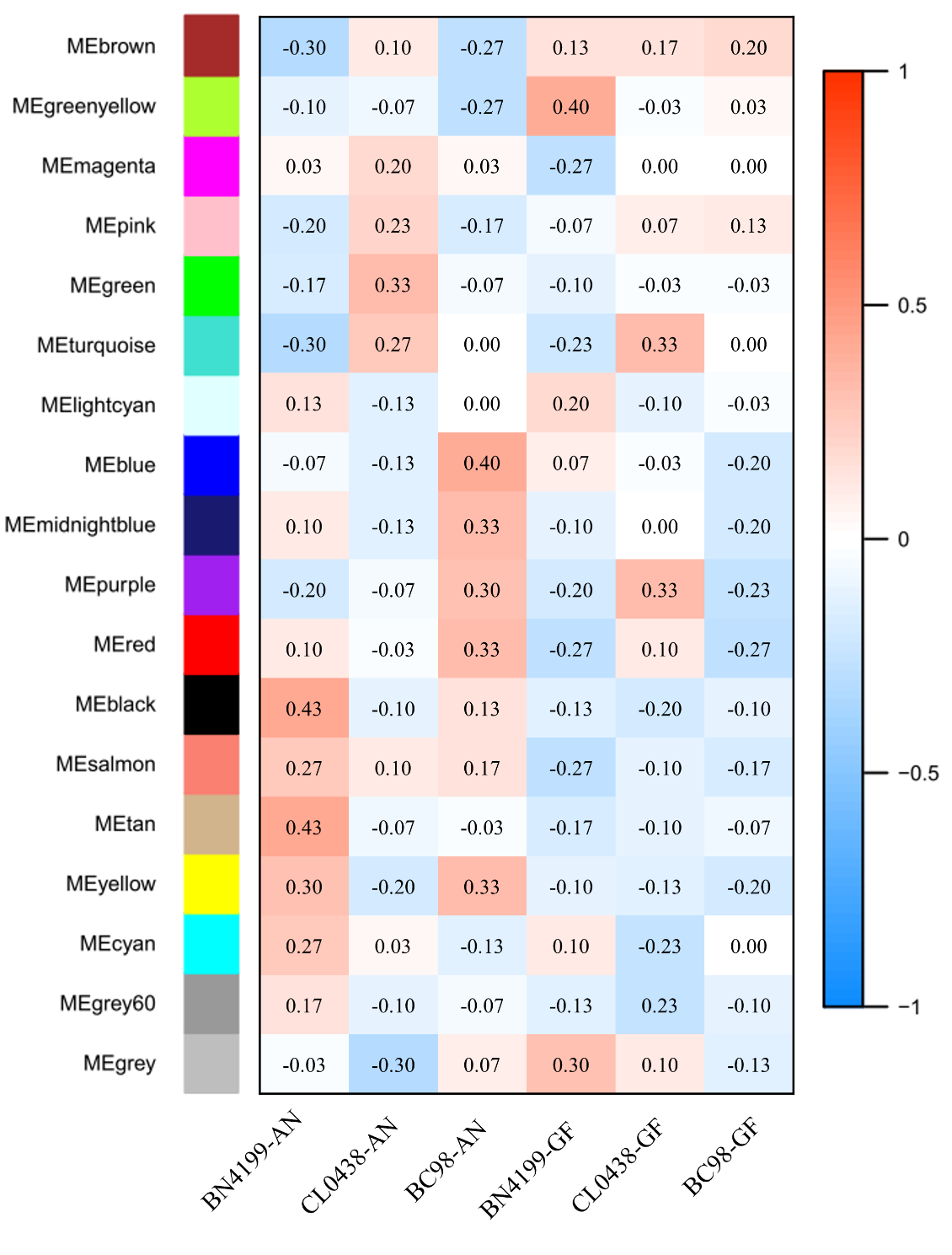


**Figure S5** Module-genotype correlation matrix across developmental stages. The horizontal axis displays sample genotypes grouped by developmental stage, while the vertical axis lists co-expression modules. AN and GF denote the anthesis stage and grain-filling stages, respectively. Positive correlations are highlighted in red, while negative correlations are highlighted in blue.


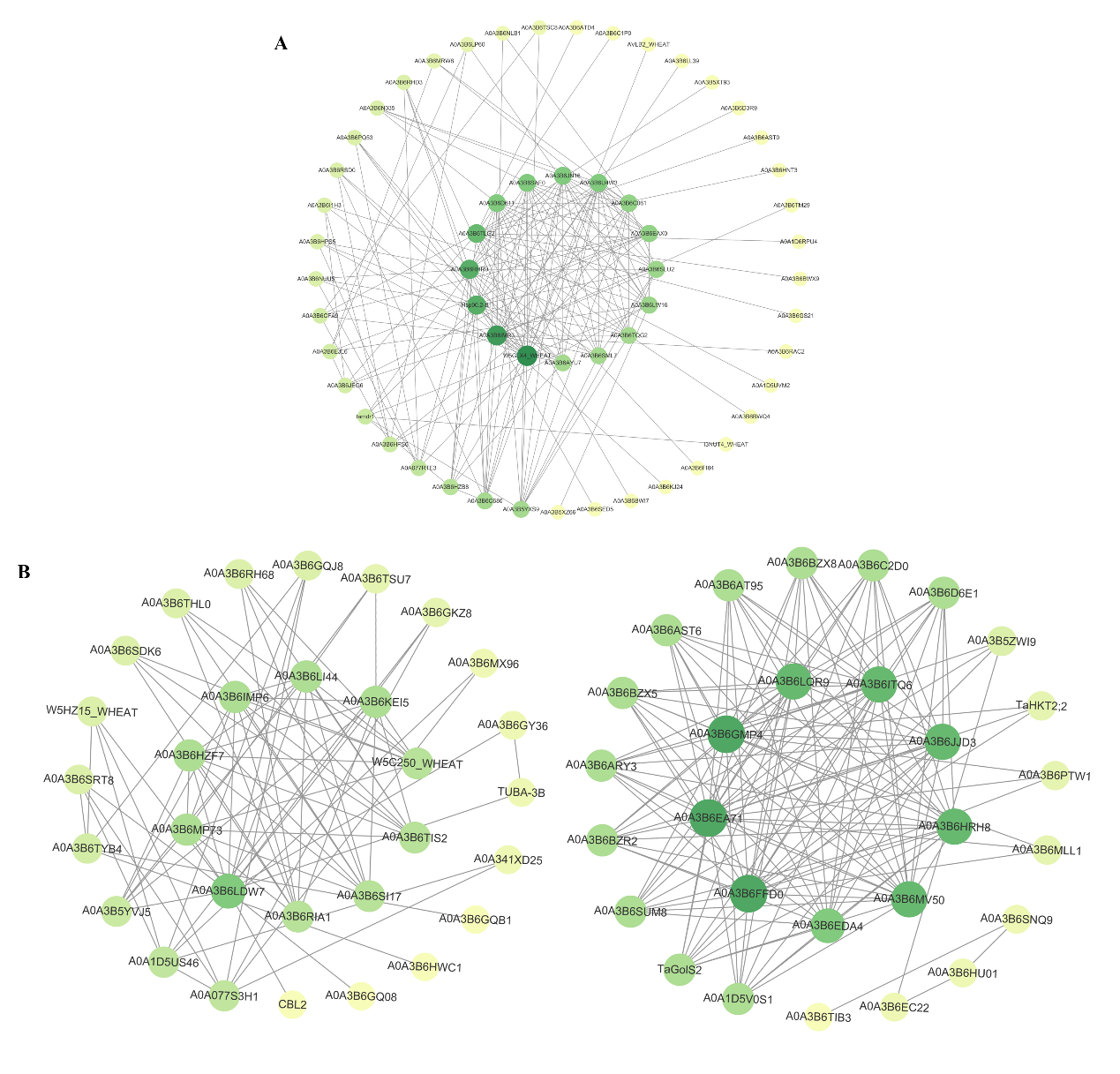


**Figure S6** Protein-protein interaction network analysis of Brown (A) and Red (B) co-expression modules. Node size reflects the number of interacting genes, with larger nodes indicating higher connectivity.


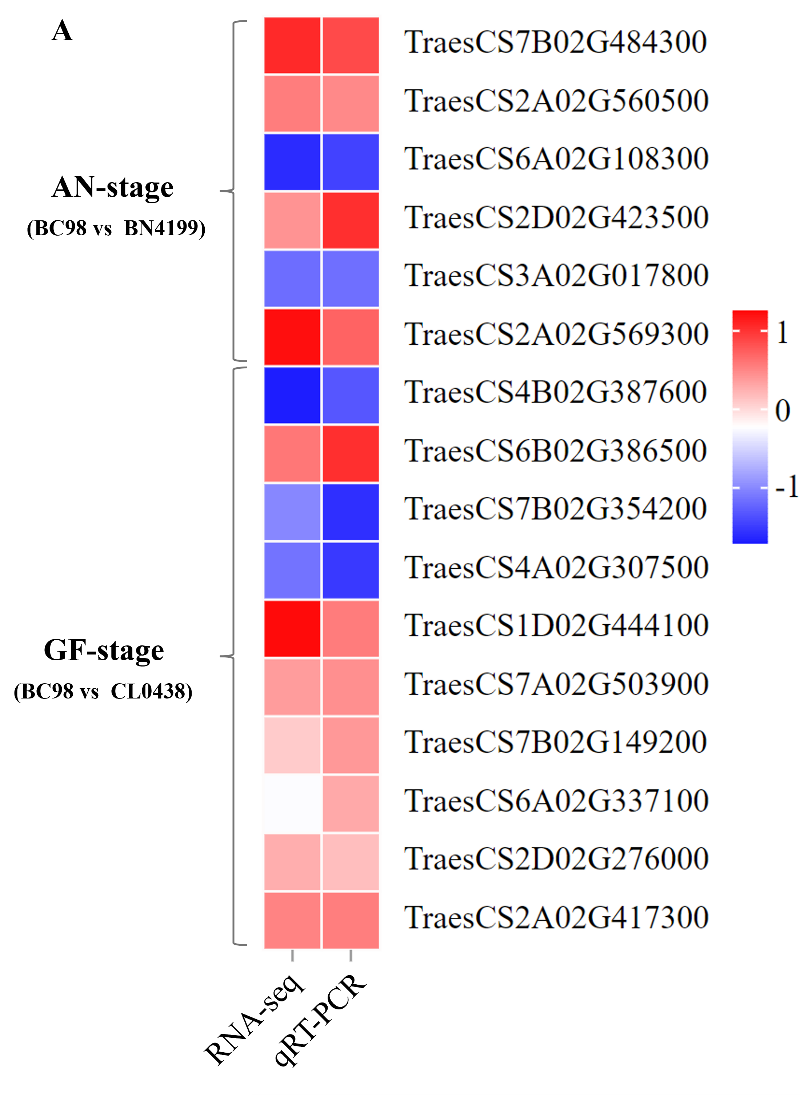


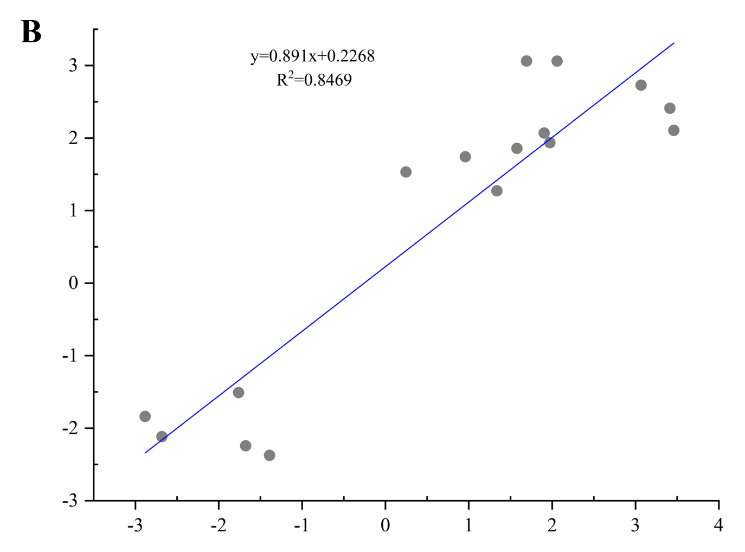


**Figure S7** Correlation analysis of selected differentially expressed genes between RNA-seq and qRT-PCR. (A) qRT-PCR validation of RNA-seq-derived gene expression during the anthesis (AN) and grain-filling (GF) stages. (B) Correlation analysis of log_2_ fold-change values between RNA-seq and qRT-PCR measurements.
